# Hybrid Homodimeric Prodrug Nanoassemblies for Low-Toxicity and Synergistic Chemophotodynamic Therapy of Melanoma

**DOI:** 10.34133/bmr.0101

**Published:** 2024-11-01

**Authors:** Peirong Xu, Fanchao Meng, Jianqin Wan, Hengyan Zhu, Shijiang Fang, Hangxiang Wang

**Affiliations:** ^1^ The First Affiliated Hospital, NHC Key Laboratory of Combined Multi-Organ Transplantation, Collaborative Innovation Center for Diagnosis and Treatment of Infectious Diseases, State Key Laboratory for Diagnosis and Treatment of Infectious Diseases, School of Medicine, Zhejiang University, Hangzhou 310003, Zhejiang Province, P. R. China.; ^2^ Department of Chemical Engineering, Zhejiang University, Hangzhou 310027, Zhejiang Province, P. R. China.; ^3^ Jinan Microecological Biomedicine Shandong Laboratory, Jinan 250117, Shandong Province, P. R. China.

## Abstract

Synergistically active nanoparticles hold great promise for facilitating multimodal cancer therapy. However, strategies for their feasible manufacture and optimizing their formulations remain lacking. Herein, we developed hybrid homodimeric prodrug nanotherapeutics with tumor-restricted drug activation and chemophotodynamic pharmacology by leveraging the supramolecular nanoassembly of small molecules. The covalent dimerization of cytotoxic taxane chemotherapy via reactive oxygen species (ROS)-activated linker yielded a homodimeric prodrug, which was further coassembled with a ROS-generating dimeric photosensitizer. The nanoassemblies were readily refined in an amphiphilic PEGylation matrix for particle surface cloaking and in vivo intravenous injection. The nanoassemblies were optimized with favorable stability and combinatorial synergism to kill cancer cells. Upon near-infrared laser irradiation, the neighboring dimer photosensitizer generated ROS, subsequently triggering bond cleavage to facilitate drug activation, which in turn produced synergistic chemophotodynamic effects against cancer. In a preclinical model of melanoma, the intravenous administration of PEGylated nanoassemblies followed by near-infrared tumor irradiation led to significant tumor regression. Furthermore, animals treated with this efficient, photo-activatable nanotherapy exhibited low systemic toxicity even at high doses. This study describes a simple and cost-effective approach to integrate multimodal therapies by creating self-assembling small-molecule prodrugs for designing a combinatorial therapeutic nanosystem. We consider that this new paradigm holds substantial potential for advancing clinical translation.

## Introduction

Skin cancer is a prevalent malignancy worldwide and is primarily caused by factors such as ultraviolet radiation and chemical carcinogen exposure, genetic variations, and compromised immune systems [1,2]. Melanoma is the most aggressive and metastatic form of skin cancer, accounting for 80% of skin cancer-related deaths, despite comprising only 5% of skin cancer cases. The World Health Organization estimates that 132,000 cases of malignant melanoma are diagnosed annually, leading to ~66,000 deaths, and its incidence continues to increase [3–5]. Surgical resection combined with chemotherapy, photodynamic therapy (PDT), and radiotherapy constitute the mainstay treatment option for melanoma [6,7]. Considering the inevitable clinical barriers of current therapies, such as severe adverse effects, high drug resistance, and radiotherapy insensitivity [2,8], developing more safe and effective therapeutic regimens for melanoma is urgent.

Nanoscale drug delivery systems that can control drug release with increased tumor-selective accumulation and reduced toxicity have been extensively studied to enhance their therapeutic index against aggressive cancers, such as melanoma [2,6,9,10]. Some formulations of therapeutic nanoparticles activated by endogenous or external stimuli have emerged, which enable tumor-restricted drug activation and pharmacology [11–14]. Among them, dimeric prodrugs, formed by ligating 2 identical or distinct drug molecules through a biologically activatable linkage [15,16], offer a cost-effective approach for creating self-deliverable nanotherapeutics. Compared with single agents, this approach provides the resultant dimers with the ability to self-assemble in aqueous solutions for intravenous injection. In this scaffold, a high drug-loading capacity can be achieved owing to the absence of additional excipients for nanoparticle assembly [17–19]. Despite these significant advantages, certain critical factors restrict their performance in vivo, leading to suboptimal efficacy. In particular, tailoring the balance between stability during blood circulation and spontaneous drug activation in target tissues or cells is important for achieving effective cancer therapy [15,19].

Taxane-based chemotherapy (e.g., docetaxel, paclitaxel, and cabazitaxel) ranks among the most widely used regimen for treating various malignancies including breast, pancreatic, peritoneal, and prostate cancers [20]. Cabazitaxel has exhibited particular potential in overcoming the chemotherapeutic resistance of current taxane owing to its diminished affinity toward P-glycoprotein. In a phase III study, cabazitaxel demonstrated considerable therapeutic benefit in extending the overall survival of patients with metastatic castration-resistant prostate cancer who were pretreated with docetaxel and refractory to docetaxel paradigm [5,21]. In addition to excipient-associated toxicity, drug intrinsic toxicity is significant, limiting the clinical efficacy and further expansion of cabazitaxel regimen to other cancers [21–23]. Our group previously endeavored to develop efficacious methods for formulating this potential agent, which led to the development of the “PUFAylation” technology, followed by small-molecule self-assembly [12,24,25] or biomaterial-assisted nanoparticle delivery [26] and dimerization-induced prodrug assembly [27]. Our previous results suggest that a rapid increase in the intracellular concentration of this agent is required, and the slow activation of cabazitaxel attenuates its efficiency as a mitotic inhibitor. These findings prompted us to optimize linker chemistry and activation approaches for novel cabazitaxel formulations to achieve robust antitumor activities at tumor sites while remaining stable in blood circulation.

**Fig. 1.**
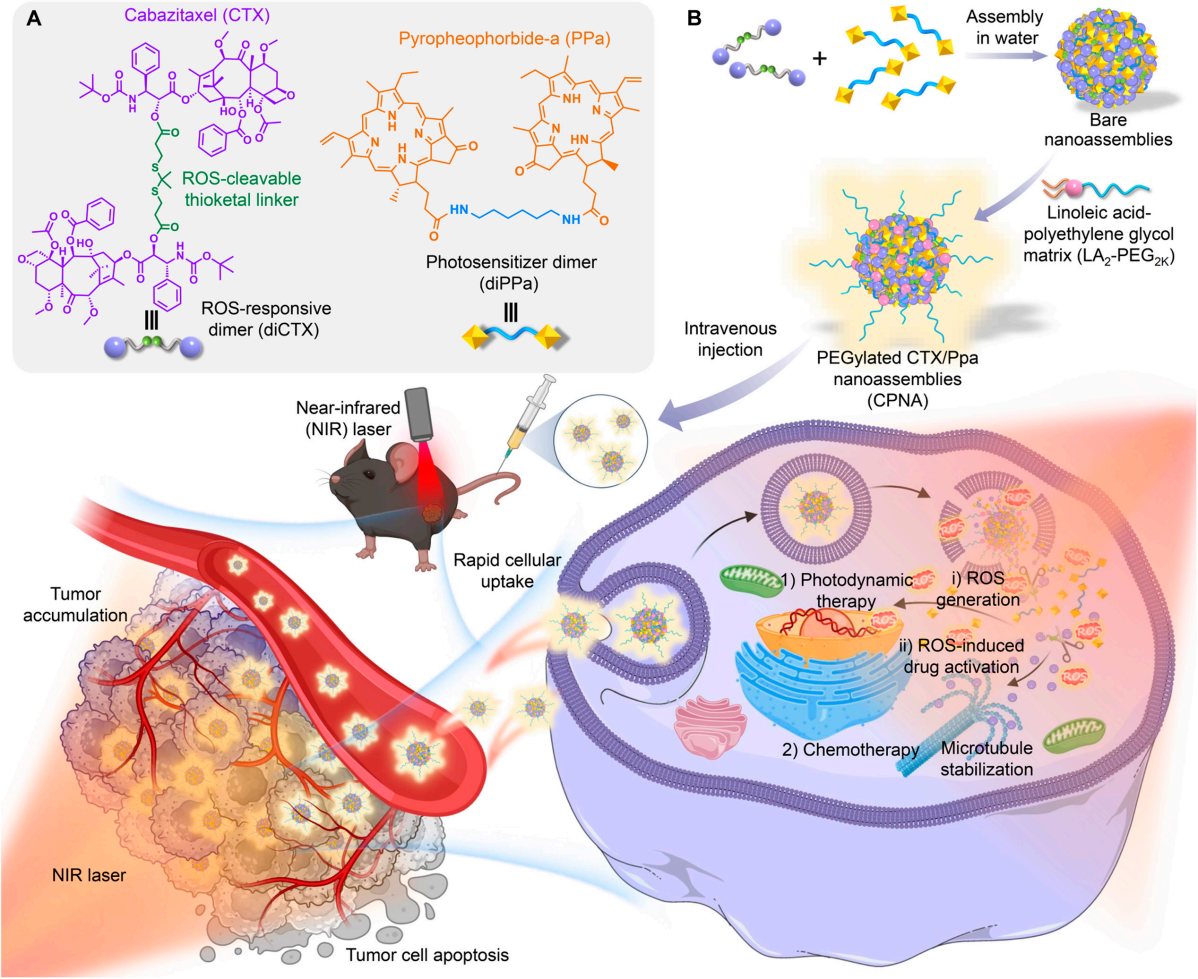
Design of supramolecular hybrid nanoassembly that responds to NIR laser irradiation for synergistic chemophotodynamic therapy. (A) Chemical structures of diCTX and diPPa. (B) Coassembly of diCTX and diPPa prodrugs in aqueous solutions results in nanostructures. Further surface cloaking of bare nanoassemblies with the LA_2_-PEG_2k_ matrix forms cabazitaxel and PPa-formulated nanoassemblies (CPNA). CPNA is intravenously injectable for therapeutic efficacy studies against melanoma in a preclinical mouse model. CPNA delivers the photosensitizer PPa to induce ROS production under 660-nm laser irradiation, triggering cabazitaxel activation from the prodrug and enabling effective synergistic chemophotodynamic therapy. Illustration created with BioRender.com.

Herein, we conceived a self-deliverable and self-activatable delivery vehicle for low-toxicity and efficacious cabazitaxel therapy against melanoma by leveraging a hybrid dimeric prodrug nanoassembly approach. Our strategy is based on the chemical ligation of cytotoxic cabazitaxel molecules via a reactive oxygen species (ROS)-responsive thioketal (TK) linker to produce a hydrolyzable dimer prodrug [dimeric cabazitaxel (diCTX)], which was further coassembled with a dimeric photosensitizer [dimeric pyropheophorbide a (diPPa)] to form cabazitaxel and PPa-formulated hybrid nanoassemblies (CPNA). Both dimeric conjugates can independently form thermodynamically stable nanoparticles that are miscible with one another at a broad range of molar ratios without the aid of additional excipients. We refined these nanoassemblies by the surface cloaking of a linoleic acid–polyethylene glycol matrix (i.e., LA_2_-PEG_2K_) for preclinical use (Fig. 1A and B). Upon near-infrared (NIR) laser irradiation, ROS generated by the neighboring diPPa not only promptly cleaved the TK linkage, leading to the rapid release of active cabazitaxel, but also facilitated PDT (Fig. 1B). In vivo studies revealed that administering optimized nanoassemblies followed by NIR laser exposure synergistically delivered potent chemophotodynamic therapy, thereby achieving highly effective antitumor activity.

## Materials and Methods

### Materials

Cabazitaxel was purchased from Jingzhu Biotechnology Co. Ltd. (Jiangsu, China). PPa was obtained from Beijing Hwrkchemical Co. Ltd. (Beijing, China). 5,5-Dimethyl-4,6-dithia-nonanedioic acid was purchased from Kailiqi Biopharma Technology Co. Ltd. (Tianjin, China). Additionally, 4-dimethylamino pyridine (DMAP), *N,N*′-diisopropylcarbodiimide (DISC), and 1,6-diaminohexane were purchased from Tokyo Chemical Industry (TCI; Shanghai, China). Indocyanine green (ICG) was acquired from Aladdin Reagents Co. Ltd. (Shanghai, China). The Cell Counting Kit-8 (CCK-8) and calcein-acetoxymethyl ester (AM)/propidium iodide (PI) dual staining kit were purchased from Dojindo China Co. Ltd. (Shanghai, China). Annexin V-FITC (fluorescein isothiocyanate) apoptosis detection kit was purchased from Vazyme Biotech Co. Ltd. (Nanjing, China). The ROS assay kit was obtained from Beyotime (Shanghai, China). DiI was purchased from US Everbright Inc. (Jiangsu, China). All other compounds and solvents were obtained from J&K Chemical (Shanghai, China) and Sigma-Aldrich, and were used without further purification. Deionized (DI) Milli-Q water (Millipore) was utilized throughout the experiments.

### Preparation of dimeric prodrug-formulated nanoassemblies

diCTX and diPPa were coassembled into drug-loaded nanoassemblies using a one-step reprecipitation protocol. Briefly, a solution of 11.29 mg of diCTX and 13.75 mg of diPPa, at a 1:2 molar ratio, dissolved in dimethyl sulfoxide (DMSO) (1 ml) was rapidly added to DI water (9 ml) under ultrasound to generate coassembled nanoparticles. Subsequently, the remaining DMSO was removed through dialysis against DI water using dialysis tubes (Spectrum, molecular weight cutoff was 7 kDa) for 24 h. To prepare PEGylated CPNA for intravenous administration, the mixture of dimeric prodrugs was blended with LA_2_-PEG_2k_ at a 10:1 weight ratio in DMSO (1 ml). This solution was subsequently added to DI water (9 ml) and subjected to the same dialysis procedure to remove DMSO. A similar procedure was used to produce other nanoassemblies with varying diCTX/diPPa ratios. The ultimate drug concentration was ascertained using reverse-phase high-performance liquid chromatography (HPLC).

### H_2_O_2_-induced cleavage of the TK linker in the diCTX prodrug

The TK linker in the diCTX prodrug is expected to be cleaved upon exposure to H_2_O_2_, resulting in the release of free cabazitaxel. In vitro hydrolysis of the diCTX conjugate in the presence or absence of H_2_O_2_ was evaluated through HPLC analysis. The diCTX conjugate (DMSO, 0.1 mg/ml, cabazitaxel-equivalent concentration) was incubated in 4 ml of a solution [phosphate-buffered saline (PBS)/acetonitrile, v/v, 4:6)] containing different H_2_O_2_ concentrations (0, 30, and 60 mM) at 37 °C. At specified time points (0.5, 1, 2, 4, 6, 8, 12, 24, 48, 72, and 96 h), 250-μl aliquots were collected, diluted with a 1:1 mixture of acetonitrile and water, and analyzed using reverse-phase HPLC at 220 nm. The HPLC was conducted at a flow rate of 1.0 ml/min, employing a gradient from 30% to 100% acetonitrile in water (0.1% trifluoroacetic acid) as the mobile phase. Quantities of compounds were determined by establishing standard curves, and the prodrug hydrolysis rate was calculated as a function of the incubation time.

### Cell lines and cell culture

Mouse breast cancer 4T1 cells were cultured in RPMI 1640 medium, while mouse melanoma B16F10 and human melanoma A375 cells were maintained in Dulbecco’s modified Eagle’s medium. All media were supplemented with 10% (v/v) fetal bovine serum (FBS), 100 units/ml of penicillin, and 100 μg/ml of streptomycin. Cells were cultured in an incubator with a humidified atmosphere containing 5% CO_2_ at 37 °C.

### Evaluation of intracellular uptake

B16F10 cells were seeded into flat-bottom 6-well plates (1.5 × 10^5^ cells/well) and cultured overnight to allow for adherence. To assess energy-dependent endocytosis, the DiI-labeled CPNA were added to the cells (100 nM DiI) and then incubated at either 4 or 37 °C for another 4 h. Cells without nanoparticle treatment were included as the control. Finally, the cells were trypsinized and washed 3 times with cold PBS, and cellular uptake of CPNA was quantified using flow cytometry.

To investigate different endocytic pathways, B16F10 cells were preincubated with specific endocytic inhibitors, including cytochalasin D (40 μM), filipin III (25 μg/ml), or chlorpromazine (10 μg/ml), at 37 °C for 30 min. Then, the cells were exposed to DiI-labeled CPNA and incubated for another 4 h. Following washing 3 times with cold PBS, cellular uptake of CPNA was quantified using flow cytometry. Cells without DiI-labeled CPNA treatment were considered the untreated groups, while cells not pretreated with any inhibitors served as the positive control.

For the mechanistic study of cellular uptake using confocal laser scanning microscopy (CLSM), B16F10 cells were plated into glass-bottom dishes (1.2 × 10^5^ cells/dish) and cultured at 37 °C for 24 h. Following the coculture with DiI-loaded CPNA (100 nM DiI) for 4 or 8 h, cells were washed 3 times with cold PBS and costained with LysoTracker Green DND-26 (Yeasen Biotechnology) for endo/lysosomes and Hoechst 33342 (blue) for nuclei for 30 min at 37 °C in the dark. Finally, cells were imaged using CLSM (FV3000, Olympus, Japan).

### Chemophotodynamic therapy-induced cytotoxicity assay in vitro

The chemophotodynamic effect of CPNA against cancer cell lines, including 4T1, B16F10, and A375 cells, was determined via the CCK-8 assay. Cells were seeded into 96-well microplates at a density ranging from 1 × 10^3^ to 4 × 10^3^ cells/well and adhered overnight at 37 °C. Subsequently, CPNA at varying concentrations in fresh culture medium was added, and the cells were subjected to an additional 12-h incubation. The culture media containing drugs were then exchanged with fresh media, followed by cell irradiation with a 660-nm laser for 5 min (300 mW/cm^2^). Cells maintained in the fresh medium were considered control groups. After an additional incubation for 60 h, cell viability was estimated using a CCK-8 assay at 450 nm with a microplate reader (Multiskan FC, Thermo Fisher Scientific). Cell viability was determined by the following formula: cell viability (%) = (absorbance of drug-treated well − absorbance of blank well)/(absorbance of untreated well − absorbance of blank well) × 100%.

### Calcein-AM/PI staining to detect cell death

The cell death resulting from chemophotodynamic toxicity was evaluated via a calcein-AM/PI costaining assay. B16F10 cells were seeded into flat-bottom 12-well plates with 6 × 10^4^ cells/well and allowed to adhere overnight at 37 °C. Various drugs, including the free drug combination, diCTX nanoassemblies, diPPa nanoassemblies, and CPNA, all at a cabazitaxel-equivalent concentration of 25 nM, were added to the cells and treated for 12 h. Subsequently, the cultured medium containing drugs in each well was removed and replaced with a fresh culture medium. The cells were then exposed to a 660-nm laser for 5 min (300 mW/cm^2^). Following another 2-h incubation, the original medium in each well was substituted with calcein-AM and PI staining solutions and incubated in the dark for 30 min. Finally, the stained cells were imaged under fluorescence microscopy (Olympus, IX71).

### Flow cytometry analysis for cell apoptosis

To assess early and late apoptotic cells resulting from chemo/PDT, 2 × 10^5^ B16F10 cells/well were seeded in flat-bottom 6-well plates and cultured to allow for adherence at 37 °C. Subsequently, cells were exposed to different drug formulations at a 100 nM cabazitaxel-equivalent concentration. After treatment with the drug for 12 h, the drug-containing culture medium was exchanged with a fresh medium. Then, the cells were irradiated with a 660-nm laser (300 mW/cm^2^) for 5 min, followed by an additional 60-h incubation. After treatment, the cells were costained with an Annexin V-FITC and PI detection kit following the manufacturer’s protocol. Finally, cell apoptosis was quantified using flow cytometry.

### Detection of intracellular ROS generation

2′,7′-Dichlorodihydrofluorescein diacetate (DCFH-DA), a fluorescence sensor that was sensitive to ROS and permeable to cells, was utilized to detect intracellular ROS generation. Briefly, B16F10 cells at a density of 1.2 × 10^5^ cells/well were seeded into flat-bottom 12-well plates and adhered overnight at 37 °C. Subsequently, different formulations containing the same cabazitaxel-equivalent concentration (25 nM) and PPa-equivalent concentration (50 nM) in a fresh culture medium were added and incubated for 12 h. Before irradiation, the culture medium in each well was exchanged with 1 ml of fresh culture medium supplemented with 10 μM DCFH-DA. After a 20-min incubation with the probe, the cells were exposed to 660-nm laser irradiation at an intensity of 300 mW/cm^2^ for 5 min (cells that were not irradiated served as a negative control) and incubated for an additional 2 h. After thorough washing, the cells were visualized under a fluorescence microscope (Olympus, IX71) or subjected to fluorescence intensity analysis using flow cytometry.

### Microtubule bundle staining assay

The immunofluorescence staining of cellular tubulin was performed to investigate the mechanism of the function of the liberated cabazitaxel. B16F10 cells were seeded in flat- and glass-bottom dishes at a density of 4 × 10^4^ cells/dish and adhered overnight at 37 °C. Following a 12-h incubation with various treatments (free drug combination, diCTX nanoassemblies, diPPa nanoassemblies, or CPNA) at a concentration of 25 nM cabazitaxel-equivalent plus 50 nM PPa-equivalent, the drug-containing medium was removed and replenished with fresh medium, following which the cells were exposed to a 660-nm laser for 5 min (300 mW/cm^2^). Following an additional 60-h incubation, the treated cells were fixed with 4% paraformaldehyde at 25 °C for 30 min. Subsequently, the cells were permeabilized with 0.5% Triton X-100-containing PBS for 2 h and blocked with 5% bovine serum albumin for 1 h at 25 °C. After washing thrice with cold PBS, the cells were immunostained with an acetyl-α tubulin (Lys40) antibody (Affinity Biosciences, USA) at a dilution of 1: 400 overnight at 4 °C and incubated with an Alexa Fluor 555 donkey anti-rabbit secondary antibody (Thermo Fisher Scientific, USA) at a dilution of 1:500 for 1 h and with Alexa Fluor 488–phalloidin (US EVERBRIGHT, China) for 40 min. All antibody diluents were prepared in PBS containing 5% bovine serum albumin. Finally, the cells were counterstained using 4′,6-diamidino-2-phenylindole for 20 min and visualized using a fluorescence microscope (Olympus, IX71).

### Animal experiments

All experimental mice were purchased from the Laboratory Animal Center of Hangzhou Medical College (Hangzhou, China) and housed in standard conditions with a 12-h light/dark cycle and free access to food and water.

### Efficacy testing against a preclinical B16F10 melanoma mouse model

Melanoma B16F10 cells (2.5 × 10^5^ cells, 100 μl) were subcutaneously implanted into the right blank of C57BL/6 mice. When the tumor volumes approached approximately 70 mm^3^, mice were randomly divided into 5 groups (*n* = 7 in each group). Mice were intravenously administered with various drugs: CPNA at low and high doses (6.0 mg/kg cabazitaxel and 7.7 mg/kg PPa, and 18.0 mg/kg cabazitaxel and 23.1 mg/kg PPa, respectively) and free drug combination (3.0 mg/kg cabazitaxel and 3.9 mg/kg PPa) for 3 successive times on days 0, 3, and 6 via the tail vein, followed by irradiation (660 nm, 600 mW/cm^2^, 10 min) at 6 h after injection. The control group was treated with saline. The tumor volume and body weight were measured and recorded every 2 d, and photographs of the tumors were taken. The tumor volume was calculated with the following formula: *V* = (*L* × *W*^2^) / 2 (*L*: length, *W*: width).

### Histopathological analysis

The tumor tissues and major organs were excised on day 12 after treatment and fixed overnight with 4% formaldehyde. After embedding in paraffin, the tissues were cut into 5-μm-thick slices. Hematoxylin and eosin (H&E; Sigma) staining was performed on the tumor and major organ sections to observe morphological changes. Tumor apoptosis was examined using the terminal deoxynucleotidyl transferase-mediated deoxyuridine triphosphate nick end labeling (TUNEL) assay with a Fluorescein-labeled In Situ Cell Death Detection Kit following the manufacturer’s protocol (Roche Applied Science). Furthermore, the proliferation of tumor cells was detected by identifying Ki67-positive cells using an anti-Ki67 antibody. The tissue slices were visualized with a fluorescence microscope (Olympus, IX71).

### Statistical analysis

All quantitative data are presented as the mean ± standard deviation. Statistical significance between measurements was estimated using Student’s *t* test, one-way analysis of variance (ANOVA) test, 2-way ANOVA test, and log-rank test. Statistical significance was denoted by *P* values, where values less than 0.05 were statistically significant. The statistical analyses were performed using GraphPad Prism 9.0 software (GraphPad software).

## Results

### Design and synthesis of photoactivatable and self-assembling dimeric prodrugs

Cabazitaxel has clinical potential as a taxane chemotherapeutic owing to its ability to overcome drug resistance induced by paclitaxel and docetaxel [28,29]. However, its poor solubility and considerable toxicity have substantially restricted clinical application [24]. Previously, we attempted to improve the therapeutic index of this agent by covalently conjugating cabazitaxel to self-assembling motifs, including polyunsaturated fatty acids and polymers [22,24]. Despite these successful demonstrations, low therapeutic activation kinetics in response to stimuli of the tumor microenvironment attenuated the efficacy of these covalently ligated prodrugs against cancer. A safer and more effective nanomedicine approach could enhance the potency of this promising chemotherapy. Therefore, a ROS-responsive diCTX was conceived and ligated via a TK linker (Fig. 1A). Intrigued by this design rationale, we further dimerized a chlorophyll-derived photosensitizer, PPa, into diPPa using a NIR light inert and noncleavable linker. Both dimer conjugates were obtained in one-step synthetic protocols (Schemes S1 and S2, Supplementary Methods). The resulting conjugates were unambiguously confirmed using nuclear magnetic resonance (NMR) spectra (e.g., ^1^H and ^13^C NMR) (Figs. S1 to S4).

### Coassembly and characterization of prodrug-loaded nanoparticles

**Fig. 2.**
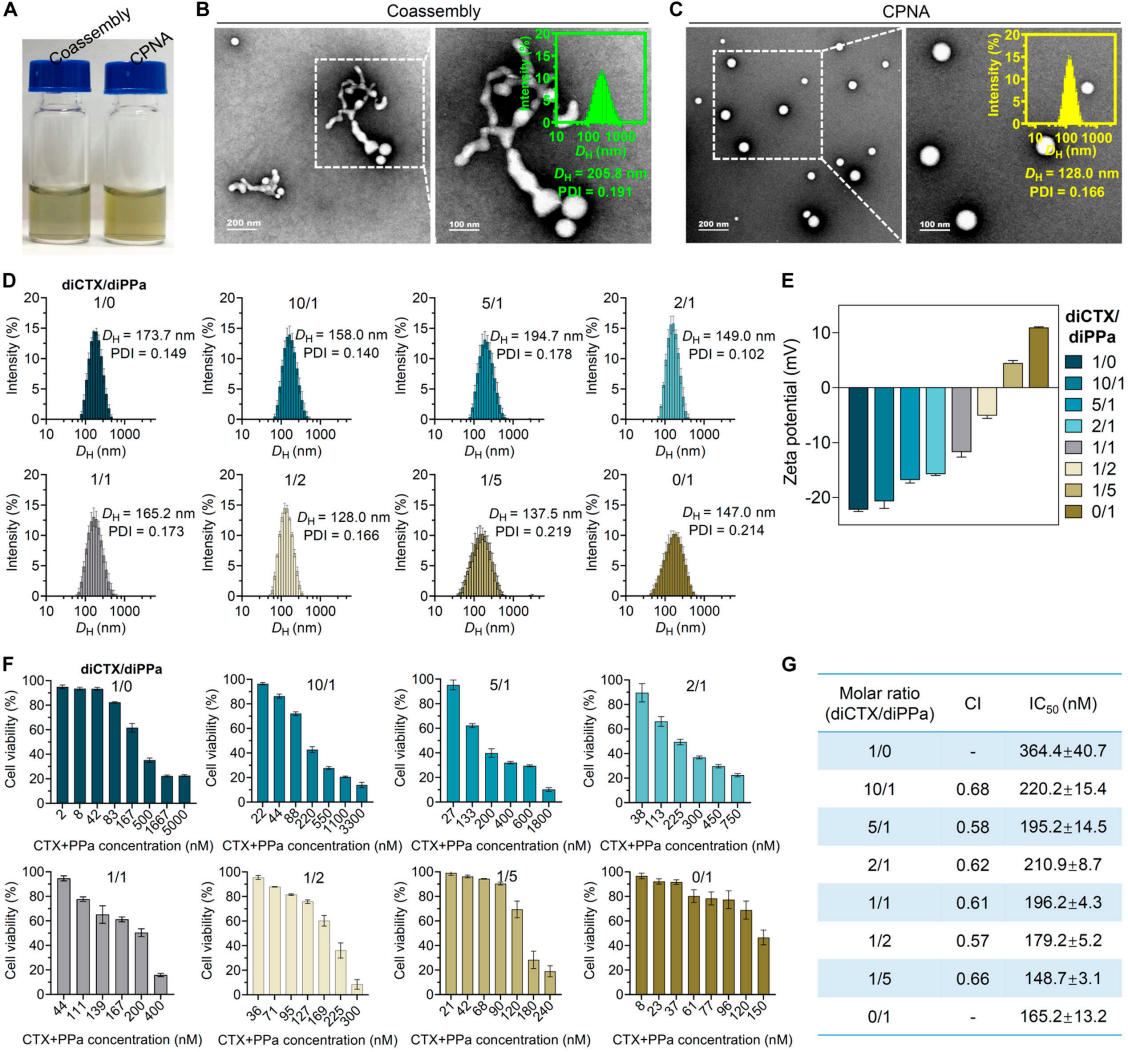
Preparation and optimization of nanoassemblies constructed from dimer conjugates. (A) Photograph and (B and C) representative TEM images and DLS of non-PEGylated (left) and PEGylated nanoassemblies (right). (D) DLS analysis and (E) zeta potentials of nanoparticles coassembled from dimer conjugates at various molecular ratios (*n* = 3). (F) Cell viability of B16F10 cells after exposure to different formulations for 12 h followed by laser irradiation (660 nm, 300 mW/cm^2^, 5 min), measured via the CCK-8 assay (*n* = 3). (G) CI and IC_50_ were extrapolated from the concentration–response curves (F). Data are presented as mean ± standard deviation.

Upon obtaining dimers, we initially followed a nanoprecipitation protocol to investigate their self-assembly behavior in an aqueous solution. Both conjugates were dissolved in DMSO and thoroughly blended, followed by the rapid injection of the mixture solution into DI water, which eventually produced a stable and transparent solution (Fig. 2A). Morphological examination using transmission electron microscopy (TEM) revealed the formation of nanosized nanostructures (Fig. 2B). However, evident irregular aggregation among these bare nanoparticles was observed, which is unfavorable for stability and in vivo therapeutic purposes. Hence, we decided to cloak these nanosuspensions using a recently developed new amphiphilic matrix omega-6 fatty acids (LA_2_-PEG_2K_) in our group. The PEGylation of the LA_2_-PEG_2K_ matrix stabilizes the nanoassemblies and may reduce clearance by the mononuclear phagocyte system (MPS) following intravenous injection. Non-PEGylated nanoparticles are rapidly cleared from circulation through mechanisms including phagocytosis by the MPS and sequestration by the liver or spleen [30]. To investigate the role of PEGylation, we varied the weight percentage of the matrix and assessed its impact on the uptake by mouse macrophages (e.g., Raw264.7 cells) [31]. Our results revealed a significant reduction in fluorescence signal levels in cells exposed to PEGylated nanoassemblies compared with those exposed to non-PEGylated counterparts (Fig. S5). Additionally, the content of PEGylation on the nanoassembly surface markedly influenced their internalization by macrophages. We previously also found that the surface cloaking of LA_2_-PEG_2K_ helps facilitate the cellular uptake of nanoparticles. Herein, we used a minimum amount of LA_2_-PEG_2K_ (10 wt%) to PEGylate the self-assembled hybrid nanoparticles. We designated these PEGylated nanoassemblies derived from diCTX and diPPa prodrugs as CPNA. Compared with bare nanoparticles, CPNA was uniformly and evenly dispersed in a spherical shape, as indicated via TEM (Fig. 2C). Dynamic light scattering (DLS) analysis revealed that CPNA exhibited a reduced *D*_H_ compared with that of the bare coassembled nanoparticles (Fig. 2B and C, insets).

diCTX and diPPa prodrugs can assemble into the CPNA scaffold at arbitrary ratios; thus, the molar ratio of CPNA was optimized for stability, particle size, and therapeutic combination index (CI). When the ratio of diCTX to diPPa was fixed at 1:2 (molar ratio), CPNA exhibited the smallest *D*_H_ (~128.0 nm) and narrow size distribution with a low polydispersity index (PDI, Fig. 2D). Furthermore, a slightly negative surface charge of −5.10 ± 0.47 mV was detected for CPNA with a molar ratio of diCTX:diPPa at 1:2 (Fig. 2E). This negative charge is advantageous for augmenting the stability of nanoparticles, as those with a pronounced positive surface charge are prone to aggregation and attract negatively charged proteins in the bloodstream, resulting in swift elimination from circulation following administration [32]. To evaluate the CI of various nanoassemblies prepared with distinct ratios, we assessed the cytotoxicity of CPNA against melanoma B16F10 cells using a CCK-8 assay. The dose-dependent cell viability is presented in Fig. 2F, and the CI along with half-maximal inhibitory concentrations (IC_50_) is summarized in Fig. 2G. In contrast to the other ratios, optimal synergistic therapeutic effect (CI = 0.57) was observed when the diCTX:diPPa ratio was 1:2. Hence, CPNA at an optimized diCTX:diPPa ratio of 1:2 was used in the subsequent experiments.

### Molecular dynamics simulations of self-assembly behavior

**Fig. 3.**
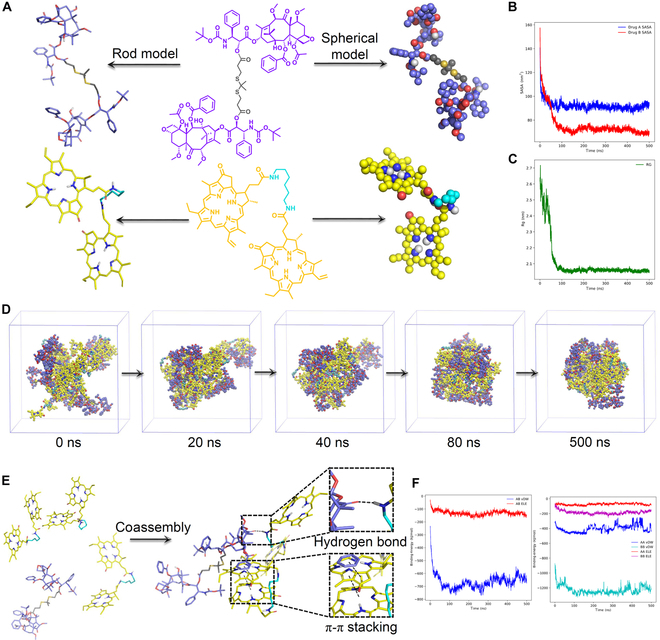
MD simulations of coassembly process. (A) Structures and molecular models of diCTX (purple) and diPPa (yellow) conjugates. Initially, 15 diCTX and 30 diPPa molecules were randomly distributed in a cubic box; aggregation occurred rapidly, maintaining a compact nanostructure throughout 500-ns simulations. The decreases of (B) solvent-accessible surface area and (C) radius of gyration values of the system indicated an aggregation tendency. (D) Key structures at different simulation times over 500 ns. (E) Multiple noncovalent interactions, including π–π stacking and hydrogen bonds, were presented in the nanoassemblies. (F) Variation in binding energy between prodrugs during the first 100 ns.

The mechanisms underlying the coassembly of these hydrophobic dimer prodrugs (Fig. 3A) were elucidated through molecular dynamics (MD) simulations. Fifteen diCTX and 30 diPPa molecules were mixed and placed in a randomly packed cubic box, with water molecules filling the remaining space. The system was then subjected to 500 ns of MD simulations. The solvent-accessible surface area and radius of gyration decreased within 100 ns, indicating that the aggregation of these molecules predominantly occurs within this timeframe (Fig. 3B and C). Upon analyzing the pivotal structures at different simulation times (Fig. 3D), a stably aggregated state was attained after 80 ns. There was no liberation of free molecules from the nanoassemblies during the entire 500-ns simulation, consistent with the variations observed in solvent-accessible surface area and radius of gyration value changes (Fig. 3B and C). We further investigated the potential molecular interactions during the assembly process. Representative interactions such as van der Waals interactions, π–π stacking, and hydrogen bonds emerged as substantial driving forces in facilitating the assembly of hydrophobic dimer conjugates (Fig. 3E) and helping stabilize the overall nanosystem. Furthermore, van der Waals interactions is predominant, as indicated by the binding energy profile during the initial 100-ns simulations (Fig. 3F). The MD simulation data revealed the ability of hydrophobic dimer molecules to self-assemble in aqueous media, further corroborating the findings from TEM and DLS analyses.

### Investigation of nanoassembly stability and ROS-triggered drug release

**Fig. 4.**
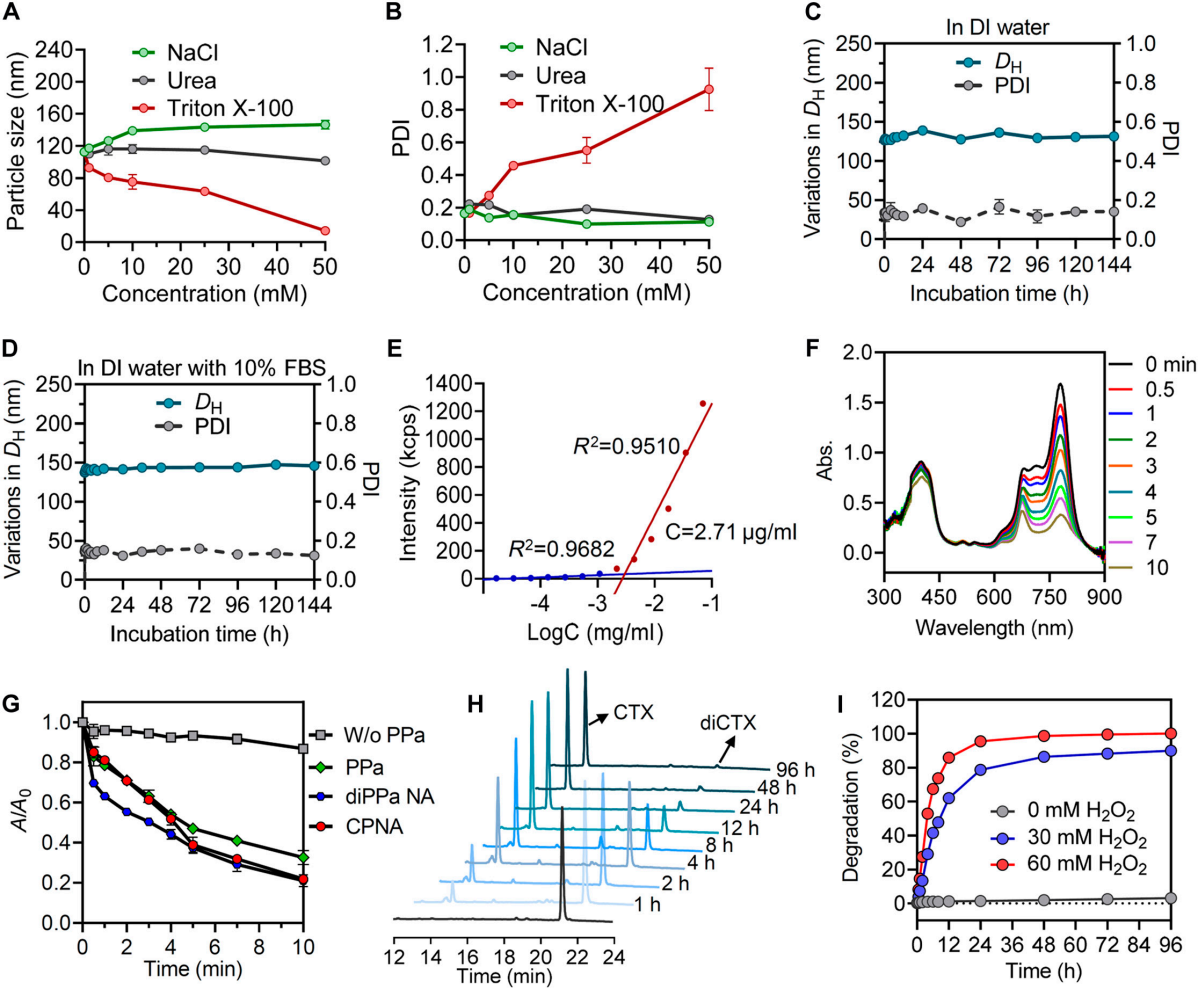
Characterization of photoactivatable CPNA. (A and B) Molecular interactions of CPNA by measuring changes in the (A) particle size and (B) PDI after the treatment with sodium chloride, urea, and Triton X-100 (i.e., 0, 1, 5, 10, 25, and 50 mM). (C and D) Stability evaluation of CPNA was analyzed through hydrodynamic diameters (*D*_H_) and PDI changes in DI water and water containing 10% (v/v) FBS. (E) Determination of the CMC of CPNA by measuring scattered light intensity. (F) Time-dependent absorption spectrum of the ROS indicator ICG decomposition in solutions with CPNA upon laser irradiation (660 nm, 300 mW/cm^2^), monitored using ultraviolet–visible spectrometry. (G) Absorption profiles of NIR light-activated ICG decrease induced by CPNA, diPPa nanoassemblies, and free PPa. Solution containing ICG with irradiation served as control. (H) Representative HPLC chromatograms of hydrolysis of diCTX prodrug to active cabazitaxel in the presence of 60 mM H_2_O_2_. (I) Degradation profiles of cabazitaxel from the diCTX prodrug at varying H_2_O_2_ concentrations over time. Data are presented as mean ± standard deviation.

The MD simulation results indicate that intermolecular forces play a crucial role in the formation and stability of coassembled nanoparticles. To further explore the dominant noncovalent interactions involved in nanoparticle assembly, Triton X-100, sodium chloride, and urea were employed to disrupt the hydrophobic and electrostatic interactions and hydrogen bonding endowed in CPNA, respectively. Upon adding the surfactant Triton X-100, the size and PDI of CPNA substantially changed, indicating the disruption of nanostructures due to hydrophobic competition (Fig. 4A and B). Conversely, adding sodium chloride and urea almost exhibited no effect on CPNA. These data suggest that hydrophobic interactions dominate the assembly process of these dimer conjugates. We further evaluated the colloidal stability of nanoassemblies in DI water or 10% FBS through DLS analysis. There were no variations in particle sizes and PDI or visible precipitates for PEGylated nanoassemblies (Fig. 4C and D), suggesting that these small-molecule dimer prodrugs were sufficiently stabilized into supramolecular nanoassemblies. However, non-PEGylated coassembled nanoparticles exhibited changes in size and PDI over time, when stored in DI water (by day 6) or in water containing 10% FBS (by day 3). Remarkably, visible precipitates from the bare nanoassemblies were observed after 7 d of storage in DI water (Fig. S6). These stability assays underscored the enhanced stability provided by the LA_2_-PEG_2K_ surface cloaking. Additionally, we assessed the stability of nanoassemblies constructed from individual prodrugs. After 24-h incubation at room temperature, visible precipitates formed in the diPPa nanoassemblies, indicating their suboptimal stability. In contrast, both the diCTX nanoassemblies and CPNA remained stable, with no signs of precipitation (Fig. S7). Upon intravenous injection, these nanoparticles tended to disintegrate into individual molecules when diluted below the critical micelle concentration (CMC). Therefore, low CMC is a critical factor for enabling intravenous injection. By quantifying scattering light intensity, we confirmed a CMC of 2.71 μg/ml for CPNA (Fig. 4E), which was lower than that of traditional micelles prepared from DSPE-PEG_2k_ (~31.82 μg/ml). These results suggest that CPNA possess favorable thermodynamic stability and a low CMC, enabling them to maintain structural integrity during blood circulation and accumulate more effectively at tumor sites.

The TK bond is prone to ROS-induced degradation, leading to the release of free cabazitaxel from prodrugs. In our nanoparticle design, the photosensitizer was integrated via molecular nanoassembly. Therefore, irradiation with a 660-nm NIR laser prompts ROS production from the neighboring photosensitizer, resulting in bond cleavage and drug activation. We compared the ROS generation ability of CPNA with that of free PPa. ICG was employed as a ROS indicator by measuring the reduction in absorbance at 779 nm, which was attributed to ICG. Following NIR laser irradiation, the ICG-derived absorption peak diminished rapidly in the solution containing CPNA (Fig. 4F). CPNA demonstrated an ability of ROS generation similar to that of solutions containing PPa or diPPa nanoparticles (Fig. 4G and Fig. S8). These data confirm that the dimerization of the photosensitizer followed by coassembly within nanoparticle scaffolds did not affect ROS-generating ability. To further assess ROS-responsive drug activation, the diCTX prodrug was exposed to H_2_O_2_. The degradation of the diCTX prodrug in the presence of 60 mM H_2_O_2_ over time was monitored using HPLC analysis (Fig. 4H). We observed the peak increase corresponding to free cabazitaxel, and conscomitantly, the peak related to the intact prodrug markedly decreased upon incubation. This result proved the activation of cabazitaxel in response to ROS. Furthermore, cabazitaxel release from the prodrug reached ~96% after 24 h of incubation with 60 mM H_2_O_2_. This TK-ligated prodrug was stable for several days without H_2_O_2_ (Fig. 4I and Fig. S9).

### Cellular uptake in cancer cells

**Fig. 5.**
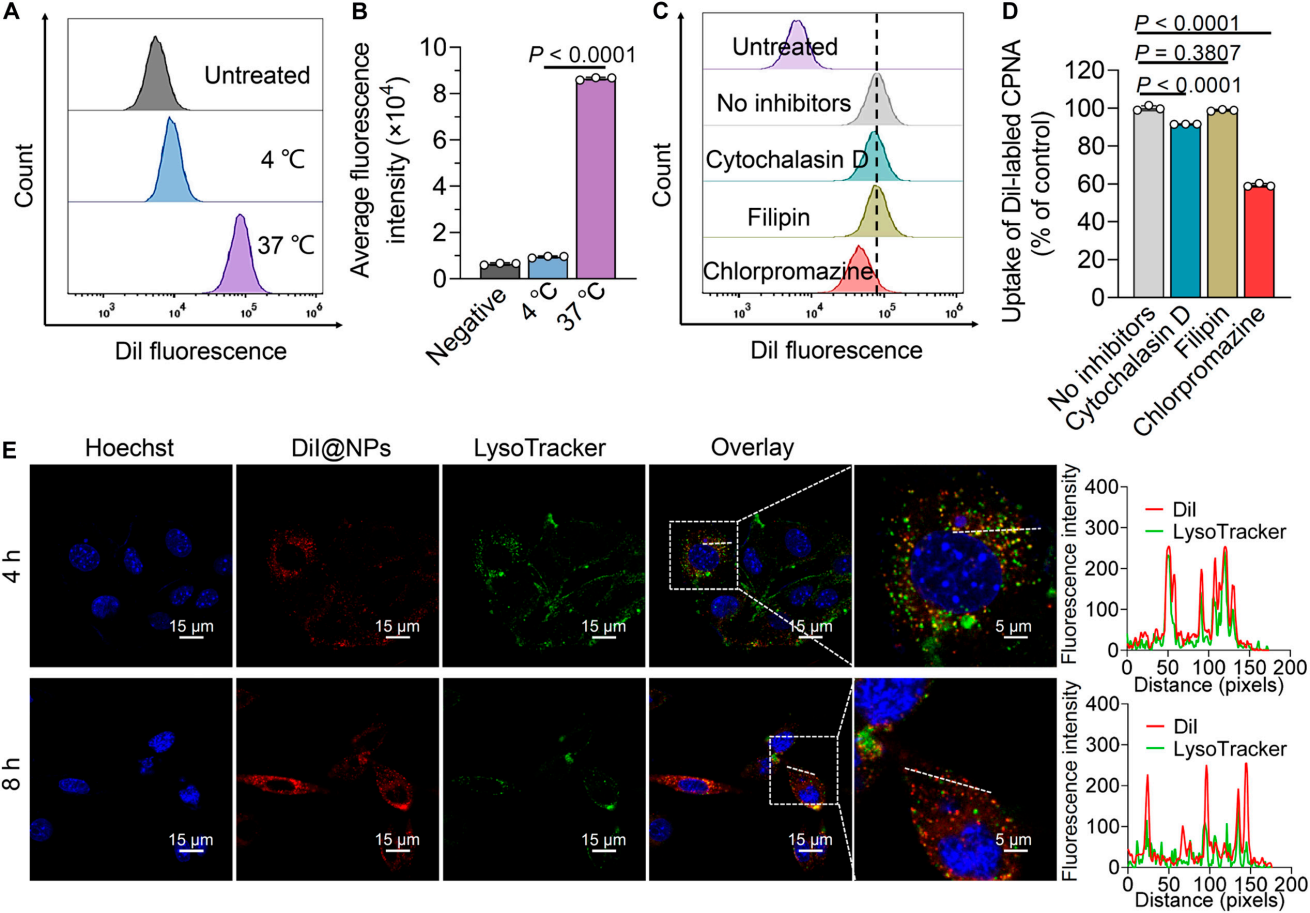
Intracellular uptake and localization of CPNA in B16F10 cells. (A and B) Flow cytometry measurement of DiI-labeled CPNA intracellular uptake after 4 h of incubation at 4 or 37 °C (*n* = 3). Untreated cells served as the negative control. (C and D) Flow cytometry analysis of nanoparticles cellular uptake pathway determined by pretreatment with endocytic inhibitors for 30 min, followed by incubation with CPNA for another 4 h (*n* = 3). (E) Nanoparticle intracellular localization observed with CLSM. Endo/lysosomes were stained with LysoTracker (green), and cell nuclei were stained with Hoechst (blue). Scale bars, 15 and 5 μm. Data are presented as mean ± standard deviation. Statistical significance was evaluated using one-way ANOVA (B and D) followed by Tukey’s multiple comparisons test.

We investigated the predominant mechanisms and pathways involved in the cellular uptake of the dimer prodrug-assembled nanoparticles. Following a 4-h incubation of fluorescent dye DiI-labeled nanoassemblies with B16F10 cells at 37 or 4 °C, the intracellular fluorescence intensity was quantified using flow cytometry. As shown in Fig. 5A and B, cells exposed to CPNA at 37 °C exhibited approximately 9-fold greater fluorescent signal compared with those incubated at 4 °C, indicating that energy-dependent endocytosis dominates the CPNA internalization. To investigate the endocytosis pathways of CPNA, we first subjected the cells to a 30-min pretreatment with various inhibitors, followed by an additional 4-h incubation with DiI-labeled nanoassemblies. These inhibitors included cytochalasin D, filipin, and chlorpromazine, targeting pinocytosis, caveolin-dependent endocytosis, and clathrin-mediated endocytosis, respectively. The cellular uptake of CPNA was determined using flow cytometry analysis (Fig. 5C and D). Clathrin-associated chlorpromazine inhibitor significantly reduced the CPNA uptake to ~59.3%, whereas cytochalasin D slightly reduced intracellular DiI fluorescence. However, treatment with filipin did not affect CPNA uptake. Hence, we can conclude that clathrin-mediated endocytosis predominantly facilitates CPNA uptake by tumor cells through the involvement of pinocytosis in nanoparticle internalization.

Intracellular distribution of CPNA was also tracked using CLSM. B16F10 cells were exposed to DiI-labeled nanoassemblies and labeled with LysoTracker Green to identify endo/lysosomal compartments. As observed in CLSM images, a substantial proportion of CPNA was transported to endo/lysosomal compartments, as indicated by their initially good colocalization with LysoTracker Green (Fig. 5E). Subsequently, the red signals from CPNA progressively increased over time and did not colocalize with LysoTracker Green at 8 h. This could be attributed to the escape of CPNA from endo/lysosomal compartments [33]. Prior studies have shown that photosensitizer-induced ROS can disrupt endo/lysosomal membranes, leading to drug redistribution from these vesicles into the cytoplasm [34–36]. Thus, this redistribution could facilitate cabazitaxel activation in the cytosol and target tubulin to stabilize microtubules.

### In vitro cytotoxicity elicited via chemophotodynamic nanotherapy in cancer cells

**Fig. 6.**
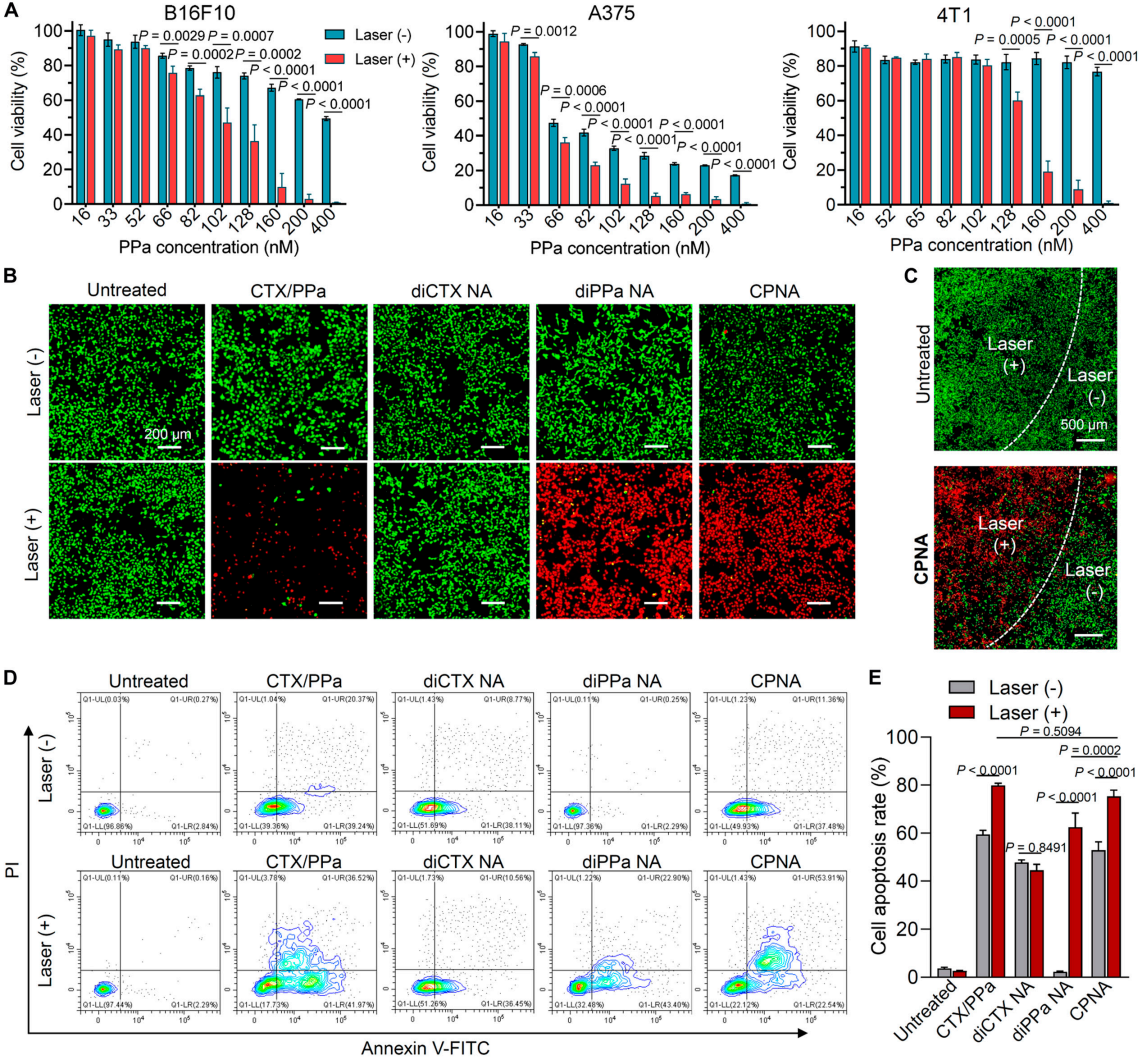
Chemophotodynamic cytotoxicity of CPNA against various cancer cell lines. (A) Evaluation of CPNA cytotoxicity in the absence or presence of laser irradiation (660 nm, 300 mW/cm^2^, 5 min) against A375, 4T1, and B16F10 cancer cells, assessed via CCK-8 assays (*n* = 4). (B) Fluorescence microscopy images revealing dead/live cell staining of B16F10 cells. Cells were treated with drugs at a cabazitaxel-equivalent concentration of 25 nM and exposed to laser irradiation (660 nm, 300 mW/cm^2^, 5min), followed by costaining with calcein-AM (green, live cells) and PI (red, dead cells). Scale bars, 200 μm. (C) Fluorescence microscopy images demonstrating locoregional photocytotoxicity induced by CPNA. B16F10 cells were treated with or without CPNA. The left side of the dotted line was irradiated with a NIR laser, while the right side was maintained in the dark. Scale bars, 500 μm. (D and E) Flow cytometry analysis of B16F10 apoptosis following different treatments at a cabazitaxel-equivalent concentration of 100 nM and PPa-equivalent concentration of 200 nM (*n* = 3). Data are presented as mean ± standard deviation. Statistical significance was evaluated using 2-way ANOVA (E) followed by Tukey’s multiple comparisons test.

Subsequently, we evaluated the in vitro cytotoxicity of CPNA against melanoma cell lines B16F10 and A375 and breast cancer cell line 4T1. Following a 12-h initial exposure to CPNA, the cell culture media containing nanoparticles were replaced with fresh media. Following NIR laser irradiation (660 nm, 300 mW/cm^2^, 5 min) and an additional 60-h incubation, cell viability was measured using the CCK-8 assay. The IC_50_ values were extrapolated from dose–response curves (Fig. 6A) and are summarized in Table S1. Without NIR laser irradiation, CPNA alone exhibited minimal cytotoxicity due to slow drug activation kinetics. When the CPNA-incubated cells were exposed to laser treatment, there was a significant reduction in cell viability. For example, laser-induced chemophotodynamic therapeutic was evident in B16F10 cells, leading to the reduction of IC_50_ values from 334.9 ± 17.8 nM to 96.9 ± 2.1 nM.

To visually validate the effects of CPNA, we performed a live/dead dual staining assay using calcein-AM and PI. Upon NIR irradiation, we observed substantial cell death as evidenced by a strong red fluorescence originating from dead cells in the CPNA-treated group as well as in the groups treated with free drug combination or diPPa nanoassemblies (Fig. 6B). Herein, the photosensitizer PPa is a critical factor in inducing cell apoptosis owing to reduced cabazitaxel cytotoxicity via chemical ligation. Therefore, in the absence of PPa or NIR irradiation, most cells remained alive. Furthermore, focused laser irradiation induced selective cell death exclusively within the NIR-irradiated region (Fig. 6C). These observations reveal that ROS production upon pinpoint NIR laser irradiation can selectively activate chemotherapy agents, leading to tumor-restricted pharmacology. Subsequently, to quantify the apoptotic cell rate following exposure to different treatments, we performed dual staining with Annexin V-FITC/PI and analysis via flow cytometry. Consistent with the results of the live/dead cell staining assay, NIR irradiation considerably increased the percentage of apoptotic cells in the treatments with free drug combination, diPPa nanoassemblies, and CPNA. CPNA exhibited a comparable potency with the combination of free drugs (75.3% versus 79.8%) (Fig. 6D and E). These data suggest that CPNA can generate efficacious intracellular ROS and effectively induce TK bond cleavage upon NIR light exposure to obtain an excellent chemophotodynamic synergistic effect.

We further compared the synergistic effects of CPNA and combined nanoparticles (i.e., prepared by physically mixing diCTX and diPPa nanoassemblies) in vitro. Following calcein-AM/PI double staining, both A375 cells (Fig. S10A) and B16F10 cells (Fig. S10B) treated with the physically mixed nanoparticles and CPNA, and then exposed to NIR laser irradiation, exhibited strong red fluorescence, indicating significant cell death induced by the chemophotodynamic therapy. To quantitatively assess their activity, treated cells were stained with Annexin V-FITC/PI and analyzed via flow cytometry. The apoptotic proportion in A375 cells treated with the physically mixed nanoparticles reached 80.97% following NIR laser exposure, while CPNA induced a comparable cytotoxic effect, with 77.14% apoptotic cells (Fig. S11A and B). Similarly, in B16F10 cells, the apoptotic percentages were 77.74% for the mixed nanoparticles and 74.34% for CPNA after NIR laser irradiation (Fig. S11C and D). These data suggest that individual diCTX and diPPa nanoassemblies, when mixed in the cell culture media, may become miscible and form hybrid nanoparticles. This is likely due to an equilibrium between small-molecule dimers and nanoaggregation, leading to the formation of coassemblies similar to CPNA. Notably, the proportion of late-stage apoptotic A375 cells [(Annexin V-FITC)^+^/PI^+^, 48.24%] was higher than that of early-stage apoptotic cells [(Annexin V-FITC)^+^/PI^−^, 28.90%] (Fig. S11D). This could be attributed to the more efficient release of cabazitaxel, facilitated by enhanced nanoparticle-mediated diCTX uptake and triggered by intracellular ROS generated from the neighboring coassembled diPPa conjugate. We also evaluated the activity of non-PEGylated bare nanoparticles. Live/dead dual staining and flow cytometry analysis showed that these nanoparticles effectively induced high levels of cell death, comparable to the effects of CPNA. These results indicate that PEGylation does not impede the synergistic cytotoxicity of CPNA.

### In vitro action mechanisms for synergistic cytotoxicity

**Fig. 7.**
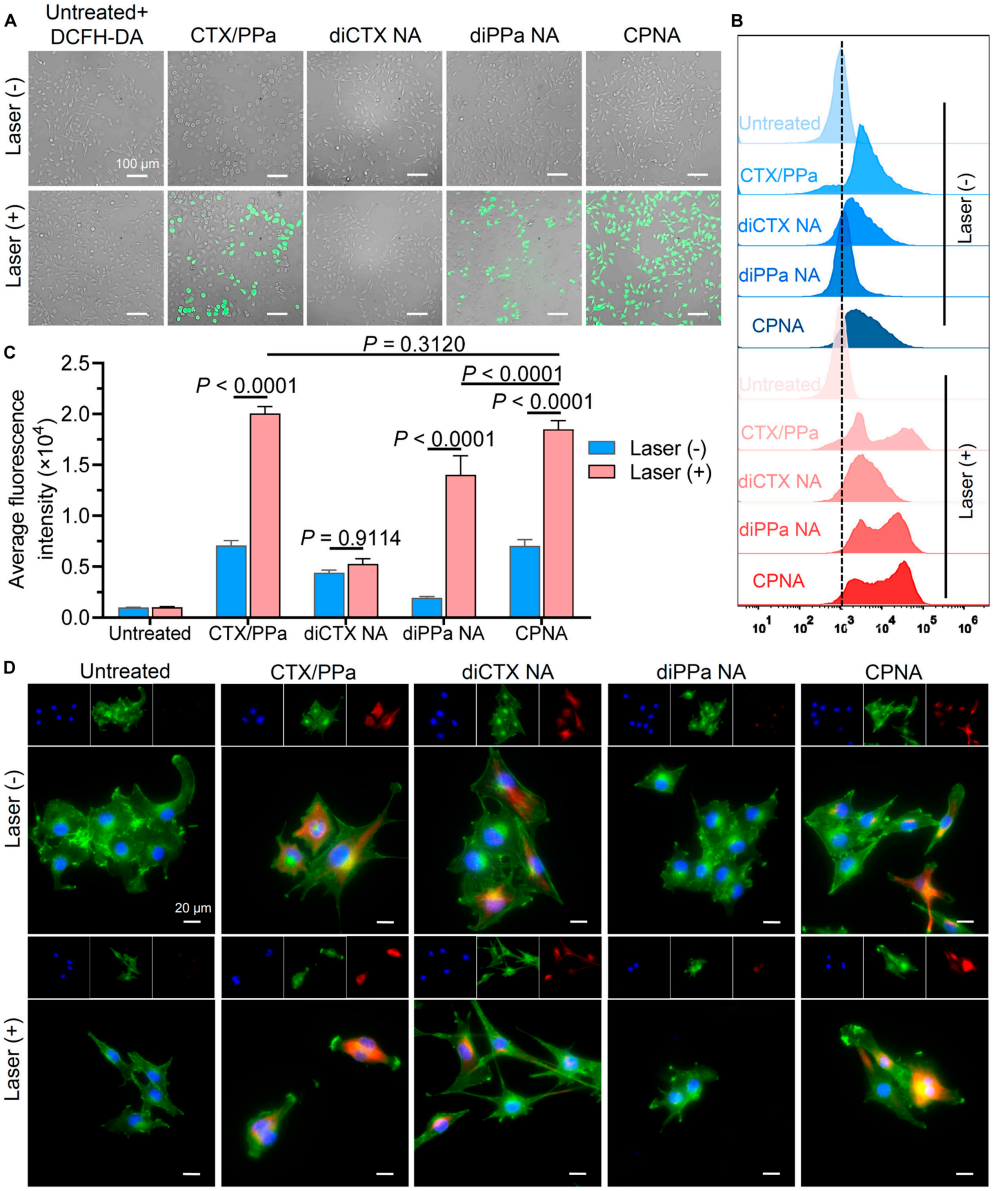
In vitro ROS generation induced by CPNA treatment with photoirradiation and mechanistic investigations of combinatorial therapy on B16F10 cells. (A to C) Intracellular ROS generation from various formulations assessed using the ROS fluorescence indicator DCFH-DA. Cells were treated with drugs at a cabazitaxel-equivalent concentration of 25 nM and then exposed to laser irradiation (660 nm, 300 mW/cm^2^, 5 min). Untreated cells served as the control. Fluorescence signals were observed under (A) a fluorescence microscope and (B and C) analyzed via flow cytometry (*n* = 3). Scale bars, 100 μm. (D) Representative fluorescence images showing increased tubulin acetylation induced by different formulations at a cabazitaxel-equivalent concentration of 25 nM, with or without laser irradiation. Red, acetyl-α-tubulin; green, F-actin; blue, nucleus. Scale bars, 20 μm. Data are presented as mean ± standard deviation. Statistical significance was evaluated using 2-way ANOVA (C) followed by Tukey’s multiple comparisons test.

We expected that once CPNA is internalized into cells and irradiated with NIR light, it would generate a substantial quantity of ROS. ROS generation can subsequently cause oxidative damage to cellular compartments and cleave the ROS-sensitive TK linker, thereby releasing active cabazitaxel to produce further cytotoxicity. Accordingly, we measured the ROS generation efficiency in B16F10 cells using the ROS generation indicator 2′,7′-dichlorodihydrofluorescein diacetate (DCFH-DA), which undergoes oxidation to produce the green fluorescent dichlorofluorescein (DCF) upon exposure to ROS in tumor cells. The results are shown in Fig. 7A to C. There was almost no green fluorescence detected in the absence of the PPa photosensitizer or 660-nm laser irradiation. However, the cells incubated with photosensitizer-containing therapies (i.e., free drug combination, diPPa nanoassemblies, or CPNA) followed by laser irradiation exhibited strong green ROS signals, as confirmed using fluorescence microscopy. Flow cytometry analysis revealed that cells exposed to CPNA followed by laser irradiation exhibited a remarkable higher intensity than CPNA without laser irradiation (Fig. 7B and C), and CPNA exhibited a comparable ROS generation efficiency with free photosensitizer. Thus, CPNA can be a promising PDT therapeutic through light-triggered intracellular ROS generation.

In addition to the PDT effect, we examined whether cytotoxicity was associated with the released cabazitaxel through immunofluorescence staining. Similar to other taxane agents, cabazitaxel binds to β-tubulin subunits of microtubules and prevents depolymerization, thereby disrupting the formation of the mitotic spindle and ultimately triggering apoptosis in fast-proliferating cancer cells. Following exposure to various drugs, the cells were incubated with acetylated α-tubulin antibody (red), phalloidin (green), and 4′,6-diamidino-2-phenylindole (blue) to label microtubules, F-actin, and nuclei, respectively. Fluorescence microscopy imaging revealed that treatment with cabazitaxel (i.e., free drug combination, diCTX nanoassemblies, and CPNA) resulted in microtubule polymerization, as evidenced by the prominent red acetyl-α tubulin fluorescence (Fig. 7D). Cells exposed to CPNA followed by laser irradiation exhibited a higher level of α-tubulin acetylation with strong red fluorescence than other treatments. However, the acetylation of α-tubulin was not observed in the diPPa-nanoassembled treated or untreated cells, regardless of laser irradiation. This immunostaining assay suggests that NIR irradiation-mediated cabazitaxel liberation from CPNA contributed to improved antitumor activity.

### Evaluation of anti-PEG antibody induced by PEGylated nanoparticles

PEG is widely utilized to enhance the circulation time and stability of nanomedicines. However, the intravenous administration of PEGylated nanomedicines can provoke the “accelerated blood clearance” phenomenon, characterized by the in vivo production of anti-PEG antibodies. These antibodies can trigger immune reactions and diminish therapeutic efficacy upon repeated dosing, presenting significant challenges for the clinical translation of PEGylated nanotherapeutics. We here evaluated the immunogenicity of CPNA by measuring the production of specific antibodies using enzyme-linked immunosorbent assay (ELISA) following the intravenous injection of CPNA (Fig. S12A). For comparison, a conventional polyethylene glycol-poly(ε-caprolactone) (PEG_2K_-PCL_2K_) matrix was included. The analysis of immunoglobulin G (IgG) and IgM antibody levels showed that the PEG_2K_-PCL_2K_ micelles induced greater immunogenicity than the PEGylated CPNA (Fig. S12B and C). After repeated dosing with PEG micelles (PEG equivalent dose of 5 μmol/kg), the highest expression of anti-PEG IgG and IgM antibodies was observed on day 7 after administration. Mice treated with PEG_2K_-PCL_2K_ micelles exhibited anti-PEG IgG levels that were 1.7-fold higher and anti-PEG IgM levels that were 2.7-fold higher than those in mice treated with CPNA on day 7. These in vivo findings provide compelling evidence that CPNA PEGylated with an LA_2_-PEG_2K_ matrix significantly attenuates the generation of anti-PEG antibodies, suggesting a low immunogenicity suitable for intravenous administration.

### In vivo biodistribution and antitumor efficacy against an A375 melanoma xenograft mouse model

**Fig. 8.**
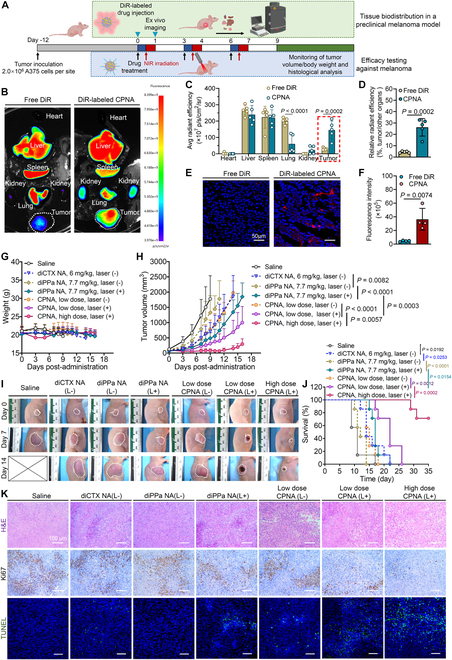
Intratumoral delivery and antitumor efficacy of CPNA in an A375 melanoma-bearing mouse model. (A) Schematic illustration of the establishment of an A375 melanoma-bearing mouse model for examining the tissue biodistribution and antitumor efficacy of CPNA. Illustration created with BioRender.com. (B) Ex vivo fluorescence images of excised major organs (heart, liver, spleen, lung, and kidneys) and tumors at 24 h after injection. (C) Quantitative evaluation of average radiant efficiency in excised major organs and tumors (*n* = 5). (D) Analysis of relative radiant efficiency ratios in tumors to other major organs (*n* = 5). (E) Representative confocal images of the DiR distribution in the tumor sections. Scale bars, 50 μm. (F) Quantitative evaluation of DiR fluorescence intensities in the tumor sections imaged by CLSM (*n* = 4). (G) Monitoring of body weight changes and (H) tumor growth curves of mice in each group (*n* = 7). (I) Representative images depicting changes in syngeneic tumors for each group on days 0, 7, and 14. (J) Mouse survival curves of different treatment groups (*n* = 7). (K) Histological analyses including H&E, Ki67, and TUNEL staining of tumor sections following treatment. Scale bars, 100 μm. Data are presented as mean ± standard deviation. Statistical significance was evaluated using 2-way ANOVA (C and H) followed by Tukey’s multiple comparisons test or Student’s *t* test (D and F) or log-rank test (J).

Melanoma, despite constituting a minority of skin cancers, is the deadliest form, necessitating effective therapeutic strategies. Taxane drugs such as paclitaxel, docetaxel, and cabazitaxel, the latter showing promise in overcoming chemoresistance, are used in melanoma therapy [37]. We explored the intratumoral delivery and therapeutic potential of CPNA in a xenograft mouse model of human melanoma. A375 melanoma cells were implanted into immunodeficient BALB/c nude mice to establish the model. The effective accumulation of the nanodelivery system within the target tumors is crucial for in vivo efficacy. To assess the tumor-targeting capability of the nanosystem in vivo, we incorporated the NIR fluorescent dye DiR into CPNA nanoassemblies to create DiR-labeled nanoparticles, visualized using In Vivo Imaging Systems (IVIS). Twelve days after subcutaneous inoculation of A375 cells, mice received intravenous injections of DiR-labeled CPNA. For comparison, free DiR dissolved in polysorbate 80/ethanol (1:1, v/v) was injected. At 24 h after injection, mice were euthanized, and their major tissues were subjected to ex vivo imaging (Fig. 8A and B). The imaging revealed predominant accumulation of free DiR in the livers, spleens, and lungs, with considerably lower signals observed in tumors. Notably, CPNA markedly increased NIR fluorescent signals in tumors while decreasing signals in the lungs. Quantitative analysis showed a 4.7-fold increase in signals for mice receiving CPNA compared to those receiving free DiR (Fig. 8C). Moreover, the fluorescence fractions of tumors relative to other major organs (Fig. 8D) exhibited greater tumor accumulation of CPNA (26.22% ± 7.67%) compared to free DiR (4.42% ± 0.79%). We further conducted cryosections of tumors and employed CLSM to observe drug accumulation. The intratumoral red fluorescence signals of CPNA exhibited significantly higher intensity compared to those of free DiR, as confirmed by fluorescence intensity analysis (Fig. 8E and F). The observed superior accumulation within tumors suggests favorable biodistribution and enhanced intratumoral delivery of CPNA, highlighting its potential efficacy in targeted drug delivery.

Encouraged by the superior in vitro cytotoxic activity and potential tumor accumulation, we subsequently explored the therapeutic efficacy of CPNA in mice bearing A375 melanoma tumors. When the tumor volume reached ~100 mm^3^, mice were randomly divided into 7 groups (*n* = 7) and received different treatment via intravenous administration: (a) saline; (b) diCTX nanoassemblies (6.0 mg/kg cabazitaxel, without NIR laser); (c) diPPa nanoassemblies (7.7 mg/kg PPa, without NIR laser); (d) diPPa nanoassemblies (7.7 mg/kg PPa, with NIR laser); (e) low-dose CPNA (6.0 mg/kg cabazitaxel and 7.7 mg/kg PPa, without NIR laser); (f) low-dose CPNA (6.0 mg/kg cabazitaxel and 7.7 mg/kg PPa, with NIR laser); (g) high-dose CPNA (18.0 mg/kg cabazitaxel and 23.1 mg/kg PPa, with NIR laser). The mice received 3 injections every 3 d, and the tumor site was subjected to NIR laser (660 nm) irradiation for 10 min at a laser power of 600 mW/cm^2^ at 6 h after administration (Fig. 8A). Body weight monitoring indicated that low-dose nanoparticle therapy combined with NIR laser irradiation did not induce body weight loss compared to the saline group. Furthermore, while mice receiving the high dose experienced a slight decrease in body weight, it quickly rebounded to normal after treatment cessation, suggesting the tolerance to this regimen in animals (Fig. 8G). Rapid tumor growth was observed in the saline group, with the volume reaching ~2,000 mm^3^ after 9 d (Fig. 8H and I). In the absence of laser irradiation, the mice receiving diPPa nanoassemblies exhibited comparable tumor growth rates to the saline-treated mice, and diCTX nanoassemblies and CPNA showed limited efficacy due to the impaired drug activation kinetics. Notably, the administration of diPPa nanoassemblies and CPNA followed by laser irradiation markedly restrained tumor growth, with CPNA exhibiting superior antitumor activity attributed to the chemotherapy efficacy of the released cabazitaxel. To explore the tolerability of CPNA and enhance its antitumor efficacy, we tripled the dosage. Strikingly, superior tumor suppression was observed in mice receiving CPNA at the 18 mg/kg cabazitaxel-equivalent dose followed by laser irradiation, resulting in a significant survival benefit during the observation period (Fig. 8J).

To further investigate the therapeutic efficacy, we conducted histopathological analysis. Tumor tissues from each group were sectioned and analyzed using H&E, Ki67 staining, and TUNEL to observe cell morphology, proliferation, and apoptosis, respectively (Fig. 8K). A substantial proportion of tumor cells exhibited pronounced damage following synergistic CPNA and laser irradiation treatment, evidenced by features such as karyopyknosis and vacuolization in H&E staining. This was in stark contrast to the densely packed tumors from saline-treated mice, showing intact chromatin and large nuclei. Ki67 staining, a specific biomarker of cell proliferation, proved that nanoparticle treatment reduced cell proliferation. We could infer that CPNA administration followed by laser irradiation effectively induced intratumoral apoptosis and suppressed tumor outgrowth. Furthermore, TUNEL, a sensitive indicator for apoptotic cancer cells, revealed extensive intratumoral apoptosis following CPNA treatment plus laser irradiation. In contrast, minimal fluorescence signals indicating apoptotic cells were observed in both the saline group and diPPa nanoassemblies without NIR laser group, consistent with the tumor growth inhibition depicted in Fig. 8H.

Considering the benefits of synergistic chemophotodynamic therapy, delivering 2 therapeutic modalities via a single nanovehicle is preferred. Therefore, we compared the in vivo activity of different delivery methods using A375 melanoma xenograft mouse model. BALB/c nude mice bearing A375 melanoma tumors (~200 mm^3^ in volume) received intravenous administration through the tail vein of CPNA or the combination of individual nanoparticles on days 0, 3, and 6. Subsequently, the tumor site was exposed to NIR laser irradiation for 10 min. We observed that both CPNA and the combined nanoparticle treatments effectively inhibited tumor growth compared to the saline control. Notably, CPNA plus NIR laser irradiation demonstrated significantly greater antitumor efficacy. As depicted in Fig. S13A and B, the average tumor volume in CPNA-treated mice was approximately 580 mm^3^ on day 10, markedly smaller than that observed with the combination of individual nanoparticles (~1,318 mm^3^). The body weight measurements revealed no significant weight loss following 3 injections of the nanoformulations, underscoring the safety of the nanotherapy (Fig. S13C). Furthermore, mice dosed with CPNA followed by laser irradiation showed improved survival (Fig. S13D). This outcome underscores the effectiveness of our design strategy: a noncovalently assembled dimer photosensitizer and cytotoxic chemotherapy within a single delivery platform. Upon delivery into tumors and subsequent NIR laser irradiation, the neighboring photosensitizer generated ROS, triggering bond cleavage to release the active drug, which synergistically enhanced the chemophotodynamic effects.

### In vivo chemophotodynamic efficacy against a B16F10 murine melanoma model without inducing systemic toxicity

**Fig. 9.**
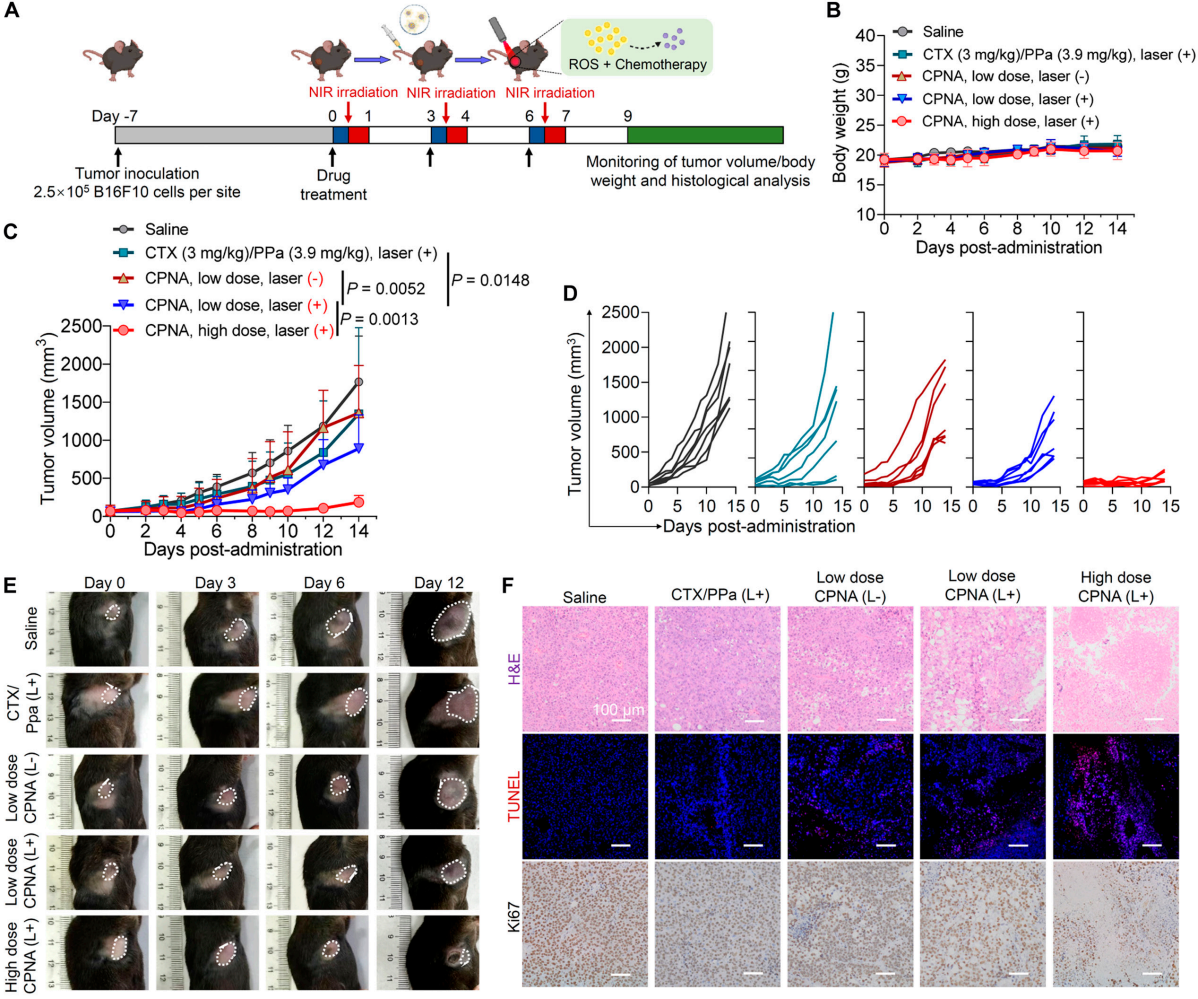
In vivo antitumor efficacy of different treatments against the melanoma mice model bearing B16F10 tumors. (A) Treatment regimen for mice with established tumors. Illustration created with BioRender.com. (B) Monitoring of body weight changes and (C) tumor growth curves of mice in each group (*n* = 7). (D) Individual tumor growth curves for mice indicated in (C). (E) Representative images depicting changes in syngeneic tumors for each group on days 0, 3, 6, and 12. (F) Histological analyses including H&E, TUNEL, and Ki67 staining of tumor sections following treatment. Scale bars, 100 μm. Data are presented as mean ± standard deviation. Statistical significance was evaluated using 2-way ANOVA (C) followed by Tukey’s multiple comparisons test.

The chemophotodynamic antitumor efficacy of CPNA was further extended to another melanoma preclinical mouse model using B16F10 cells. C57BL/6 mice bearing B16F10 melanoma tumors (~70 mm^3^ in volume) were intravenously administered with CPNA (low dose at 6.0 mg/kg cabazitaxel and 7.7 mg/kg PPa and high dose at 18.0 mg/kg cabazitaxel and 23.1 mg/kg PPa) through the tail vein on days 0, 3, and 6 (experimental protocol shown in Fig. 9A). Owing to the high toxicity observed in our previous studies, a relatively low dose of free drug combination (3.0 mg/kg cabazitaxel and 3.9 mg/kg PPa) was included for comparison. At 6 h after administration, the tumor region was irradiated with NIR laser (660 nm) at a power of 600 mW/cm^2^ for 10 min. The tumor-focused irradiation was anticipated to induce ROS production through diPPa coassembly in the nanoassemblies, consequently triggering the release of cabazitaxel and resulting in an effective PDT effect. The body weight curve revealed no signs of weight loss, even after a 3-fold increase in drug dosage, indicating the safety and tolerability of CPNA treatment (Fig. 9B). During the observation period, the tumor grew aggressively in the saline group, and the volume reached >2,000 mm^3^ after 14 d. The combination of free cabazitaxel and PPa with laser irradiation only partially delayed the tumor growth, most likely because of its aggressive nature. In addition, we found that the treatment of the nanoassemblies alone without laser irradiation exhibited comparable efficacy with that of free drugs. However, the antitumor activity was potentiated by additional exposure to laser irradiation. Encouraged by increased tolerability, we attempted a dose intensification in mice. The results reveal that treatment with a high dose of CPNA at 18 mg/kg of cabazitaxel-equivalent dose plus laser irradiation outperformed other treatments in terms of efficacy, leading to substantial tumor shrinkage throughout the whole observation period (Fig. 9C to E). We further conducted histopathological analysis of the treatment outcomes using H&E, TUNEL staining, and Ki67 immunohistochemistry (Fig. 9F). Consistent with the tumor growth patterns observed in the A375 melanoma model and the tumor progression curves in Fig. 9C, we observed a similar trend in this model, with the combination of CPNA and laser irradiation eliciting marked intratumoral apoptosis.

We performed histological analysis to evaluate potential toxicity in the organs of mice receiving CPNA therapy. The major organs harvested from each treatment group were subjected to H&E staining for careful investigation. As shown in Fig. S14, the combination therapy of free drugs plus laser irradiation caused severe liver damage, evident from the abundance of liver necrosis and cavities. Partial glomerular atrophy and necrosis were also observed in mouse kidneys when treated with free drugs. Conversely, all livers and kidneys excised from the mice administered with CPNA nanotherapy at low or high doses displayed similar histological patterns to those of saline-treated mice, regardless of laser irradiation. These data evidence that chemical ligation followed by a nanoassembly approach has transformed this potent cytotoxic drug into a more tolerable dosing regimen, enhancing its antitumor efficacy while alleviating systemic toxicity.

### In vivo safety assessment of CPNA in ICR mice

**Fig. 10.**
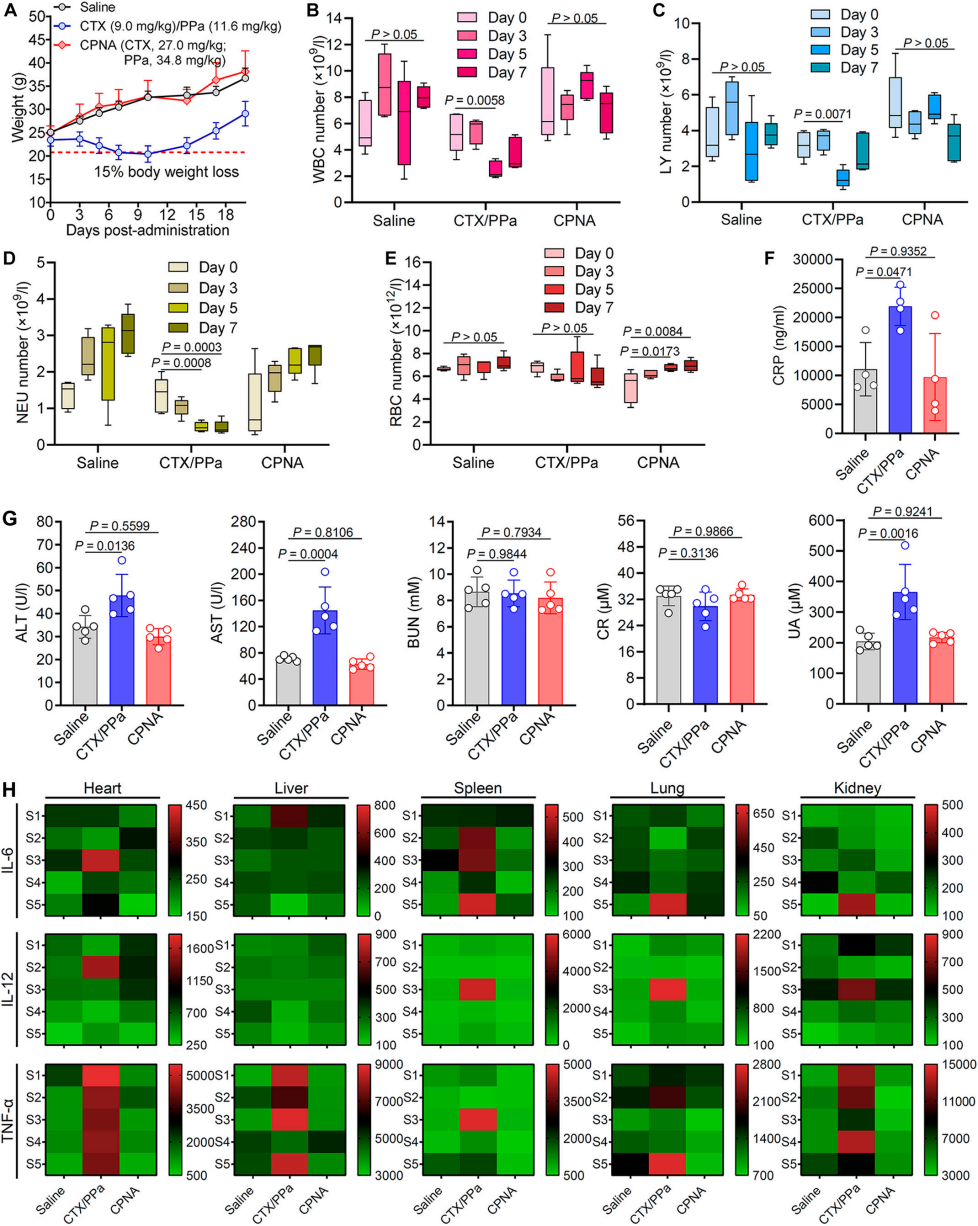
Comprehensive toxicological profile of CPNA in ICR after administration. (A) Body weight changes of ICR mice over 20 d after administration, delineating the effects of saline, a free drug combination (cabazitaxel at 9 mg/kg and PPa at 11.6 mg/kg), and CPNA (27.0 mg/kg cabazitaxel and 34.8 mg/kg PPa) (*n* = 5). (B to E) Longitudinal hematological analysis showing WBCs, LYs, NEs, and RBCs measured on days 0, 3, 5, and 7 after administration of various drug formulations (*n* = 5). (F) Quantification of serum CRP levels in treated mice on day 8 following 3 injections (*n* = 4). (G) Liver and kidney function profiles evaluated by serum biochemical markers including ALT, AST, BUN, CR, and UA on day 8 after treatment (*n* = 5). (H) Heat maps depicting the cytokine milieu in major organs, quantifying IL-6, IL-12, and TNF-α concentrations through ELISA on day 8 after treatment (*n* = 5). Data are presented as mean ± standard deviation. Statistical significance was evaluated using one-way ANOVA (B to G) across treatment groups (B to G) followed by post hoc Tukey’s multiple comparisons test.

Cabazitaxel, commercially known as Jevtana and formulated with Tween 80, has been US Food and Drug Administration approved for treating metastatic castrate-resistant prostate cancer (mCRPC). However, its maximum tolerated dose for a single injection is markedly lower (25 mg/m^2^) than those of paclitaxel (175 mg/m^2^) and docetaxel (75 mg/m^2^), underscoring its pronounced systemic toxicity. Inspired by the promising tolerability observed in murine models, we finally assessed the safety profile of our nanoassemblies through body weight monitoring, complete blood counts, serum biochemical analysis, and histological ELISA analysis. Healthy ICR mice were intravenously administered either a free drug combination (9.0 mg/kg cabazitaxel and 11.6 mg/kg PPa) or CPNA at a 3-fold higher dose (27.0 mg/kg cabazitaxel and 34.8 mg/kg PPa), with saline serving as the control. After 3 treatment doses, the free drug combination group exhibited a notable decline in body weight (~15% loss by day 7), whereas the CPNA group maintained body weight comparable to the saline group, demonstrating at least a 3-fold enhancement in tolerability (Fig. 10A).

Hematological assessments were performed to gauge potential toxicity, with a focus on parameters such as neutrophils (NEs), white blood cells (WBCs), lymphocytes (LYs), and red blood cells (RBCs). The free drug combination group exhibited significant reductions in these counts, indicative of leukopenia and neutropenia, common side effects of cabazitaxel (Fig. 10B to E). Conversely, the CPNA-treated mice showed no statistically significant alternation in these counts compared to the saline-treated mice, even at the elevated cabazitaxel-equivalent dose of 27 mg/kg. Serum biochemical analyses further elucidated drug-induced inflammation and tissue damage. The free drug combination led to elevated levels of C-reactive protein (CRP), a marker of inflammation, whereas CPNA-treated mice displayed CRP levels akin to the saline group (Fig. 10F). Additionally, hepatorenal function parameters, including alanine aminotransferase (ALT), aspartate aminotransferase (AST), blood urea nitrogen (BUN), creatinine (CR), and uric acid (UA), showed no significant deviations between the saline-treated and CPNA-treated groups, suggesting the absence of hepatic or renal toxicity (Fig. 10G). In stark contrast, mice administered intravenously with the free drug combination experienced notable increases in ALT, AST, and UA levels, indicating pronounced hepatic and renal toxicity.

To further assess the inflammatory response, we quantified typical cytokines/chemokines [e.g., interleukin-6 (IL-6), IL-12, and tumor necrosis factor-α (TNF-α)] in major organs using ELISA. Organs of mice treated with the free drug combination displayed increased inflammation, particularly TNF-α in the heart, liver, and kidneys, and elevated IL-6 levels in the spleen, compared to the saline group. In contrast, CPNA treatment resulted in minimal alternations in these cytokines/chemokines, indicating reduced systemic inflammation and immunotoxicity (Fig. 10H). Overall, these findings corroborate the efficacy of our nanoassembly approach in mitigating cabazitaxel toxicity, thereby establishing the favorable safety profile and enhanced drug tolerance of using the CPNA platform.

## Discussion

There is an urgent need to continue searching for high-efficiency and low-toxicity chemotherapy modalities for cancer treatment. Nanoparticle-based drug formulation technologies and medicines hold great potential to improve the therapeutic index of numerous anticancer drugs [38]. However, several important challenges remain in translating these nanotechnological platforms into clinical applications. Specifically, complexity, reproducibility, and safety concerns are major factors deterring their further clinical translation [39]. The strategy of dimerization-induced drug conjugate assembly might be a promising approach to address such issues. There are some pioneering examples of dimeric prodrug nanoassemblies based on small-molecule anticancer therapeutics (e.g., paclitaxel, docetaxel, doxorubicin, and SN38) under preclinical development [17]. These delivery systems have some advantages, including high drug-loading capacity, easy compound synthesis and nanoparticle manufacture, high batch-to-batch reproducibility, and minimal excipient-associated toxicity [40]. Despite these significant advances, the balance between stability in blood circulation and drug activation in target cancer cells is crucial for effective treatment [14,15,41]. Efforts to rationalize the linker chemistry and activation strategy are warranted to improve the performance of these dimer prodrug-based nanotherapeutics.

PDT uses light-absorbing photosensitizers to convert oxygen into ROS under laser irradiation, usually NIR light, to kill cancer cells [42,43]. As a minimally invasive treatment modality, PDT has exhibited spatiotemporal selectivity, high efficacy, and low toxicity [44]. In addition, PDT has been exploited as a therapy in clinical practice, including melanoma therapy. Moreover, numerous preclinical and clinical results support the notion that PDT in a rational combination with standard chemotherapy can achieve better outcomes than monotherapy [45–47]. In addition to photocytotoxicity or combinational effect, NIR light can be used as an exogenous source to control the pharmacological actions of a given agent [11,12,48]. However, administering a simple mixture of solution-based free therapeutics generally exhibits poor target selectivity, such as in tumor lesions and suboptimal synergy, owing to several challenges: (a) differences in the pharmacokinetics and biodistribution of structurally different agents, (b) uncontrollable approach to intracellular targets with desired drug concentrations and ratios, and (c) “always-on” toxicity of multiple drugs that imposes an extra burden on patients.

To address the issues of using small-molecule drug administration and combination, we developed a conceptually new hybrid supramolecular assembly of homodimeric prodrugs for synergistic cancer therapy. As a proof-of-principle demonstration, a photosensitizer (i.e., PPa) and potent cytotoxic chemotherapeutic drug (i.e., cabazitaxel) were covalently ligated to generate diPPa and diCTX, respectively. As a taxane agent, cabazitaxel binds to tubulin, thereby stabilizing microtubules and triggering cell death [5,49]. Therefore, it is necessary to liberate active cabazitaxel to exert its cytotoxicity. We accordingly designed a ROS-cleavable TK linker for homogeneous generation of diCTX. On the other hand, the photodynamic activity and ROS production for the photosensitizer PPa do not require the release of free PPa. We therefore ligated PPa via a NIR light inert and noncleavable linker. Unlike free agents that cannot self-assemble, dimerization triggered these dimer molecules to form colloidal stable nanoparticles for systemic injection and preclinical studies. In addition, this facile and cost-effective protocol enables nearly quantitative entrapment efficiencies and exceptionally high drug-loading capacities (>89%). To facilitate intracellular ROS generation, a dimer photosensitizer was incorporated, which spontaneously produced a substantial amount of ROS under NIR laser irradiation. Thus, this photoactivatable platform can synchronize NIR light-induced photocytotoxicity and enable light-triggered on-demand delivery of cytotoxic agents, offering a synergistic approach for cancer treatment. In preclinical aggressive A375 and B16F10 mouse models, we observed durable tumor regression following intravenous nanotherapeutic injection followed by pinpoint laser irradiation. In addition, we found that the CPNA therapy was well tolerated in animals, as supported by stable body weight growth during the high-dose treatment.

In summary, we successfully created a self-deliverable NIR light-responsive platform for the synergistic delivery of multiple therapeutic modalities to enhance the effect of melanoma therapy. In contrast to numerous reported drug combinations and delivery systems, this strategy is more convenient. These dimer derivatives can be readily synthesized from commercially available reagents via a one-step reaction protocol, and the manufacture of nanoparticles is straightforward. This study has implications for the clinical development of therapeutic anticancer nanomedicines.

## Ethical Approval

The mouse experiments were conducted in compliance with the guidelines outlined in the National Institute Guide for the Care and Use of Laboratory Animals. The experimental procedures were approved by the Ethics Committee of the First Affiliated Hospital, Zhejiang University School of Medicine.

## Data Availability

The datasets used and/or analyzed during the current study are available from the corresponding author on reasonable request.
