# Supplementary material for: Hybrid Homodimeric Prodrug Nanoassemblies for Low-Toxicity and Synergistic Chemophotodynamic Therapy of Melanoma
Source: Biomater Res. 2024 Nov 1;28:0101. doi: 10.34133/bmr.0101 (PMC11529783; doi:10.34133/bmr.0101)
Supplement: Supplementary 1 — Supplementary Methods Table S1 Figs. S1 to S14 [file bmr.0101.f1.zip › Supplementary Information.pdf]

Supplementary Information

Hybrid homodimeric prodrug nanoassemblies for low toxicity and synergistic chemo-photodynamic therapy of melanoma

Peirong Xu[1,2], Fanchao Meng[1], Jianqin Wan[1], Hengyan Zhu[1], Shijiang Fang[2], and Hangxiang Wang[1,3]*

[1] The First Affiliated Hospital, NHC Key Laboratory of Combined Multi-Organ Transplantation, Collaborative Innovation Center for Diagnosis and Treatment of Infectious Diseases, State Key Laboratory for Diagnosis and Treatment of Infectious Diseases, School of Medicine, Zhejiang University, Hangzhou 310003, Zhejiang Province, P. R. China

[2] Department of Chemical Engineering, Zhejiang University, Hangzhou 310027, Zhejiang Province, P. R. China

[3] Jinan Microecological Biomedicine Shandong Laboratory, Jinan 250117, Shandong Province, P. R. China

Corresponding Author: Hangxiang Wang, E-mail: wanghx@zju.edu.cn

Supplementary Methods

Characterization of synthesis

All reactions were conducted under anhydrous conditions. Thin-layer chromatography was conducted on silica gel 60 F254-precoated aluminum sheets (Merck) and visualized using fluorescence quenching. Column chromatography on neutral silica gel (Qingdao Haiyang Chemical Co., Ltd) was employed for compound purification. Proton nuclear magnetic resonance (^{1}H NMR) spectra were recorded using a Bruker 400 spectrometer at 400 MHz in CDCl3. Chemical shifts were calibrated to the residual solvent peak or tetramethylsilane (= 0 ppm). The multiplicity abbreviations are: s = singlet, d = doublet, t = triplet, q = quartet, m = multiplet, and dd = double doublet.

Synthesis of dimer cabazitaxel prodrug

Cabazitaxel (400 mg, 0.48 mmol, 2.2 eqv.) and 5,5-dimethyl-4,6-dithia-nonanedioic acid (55.0 mg, 0.22 mmol, 1.0 eqv.) were dissolved in dry dichloromethane (DCM, 4 mL). To this solution, 4-dimethylamino pyridine (DMAP) (87.7 mg, 0.72 mmol, 3.3 eqv.) and 1-(3-dimethyl aminopropyl)-3-ethyl carbodiimide (EDC) (111.4 mg, 0.72 mmol, 3.3 eqv.) was added. The reaction mixture was stirred at 45ºC overnight to facilitate dimerization. After confirming the completion of the reaction by thin-layer chromatography (TLC), the mixture was cooled to room temperature and then extracted with DCM. The residue was dissolved in DCM and subsequently washed with 5% aqueous citric acid, saturated aqueous $NaHCO_3$, and brine. The organic layer was dried over anhydrous Na_2SO_4, filtered, and then evaporated under vacuum. Finally, the crude residue was purified by column chromatography (DCM: ethyl acetate = 2:1) on silica gel, yielding the dimer conjugate diCTX (245.9 mg, 59.9%).

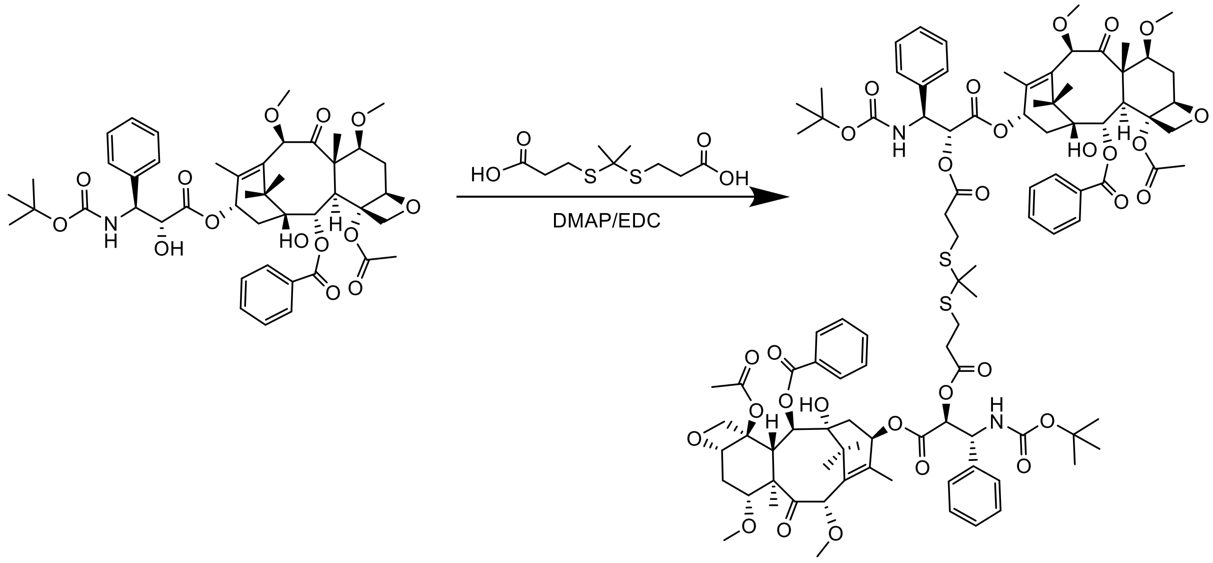

1

.

^{1}H NMR (400 MHz, Chloroform-*d*) δ 8.12-8.10 (d, 4H, *J* = 7.6), 7.63-7.59 (t, 2H, *J* = 7.4), 7.52-7.48 (t, 4H, *J* = 7.6), 7.42-7.38 (m, 4H), 7.33-7.30 (m, 6H), 6.29-6.25 (br, 2H), 5.66-5.64 (d, *J* = 7.0, 2H), 5.48-5.44 (m, 4H), 5.35 (s, 2H), 5.01-4.99 (d, *J* = 9.5, 2H), 4.82 (s, 2H), 4.33-4.31 (d, *J* = 8.4, 2H), 4.16-4.18 (d, *J* = 8.5, 2H), 3.92-3.87 (dd, *J* = 10.7, 6.4, 2H), 3.85-3.84 (d, *J* = 7.0, 2H), 3.43 (s, 6H), 3.30 (s, 6H), 2.81-2.66 (m, 8H), 2.65-2.56 (m, 2H), 2.45 (s, 6H), 2.36-2.11 (m, 4H), 2.00 (s, 6H), 1.85-1.74 (m, 2H), 1.71 (s, 6H), 1.62 (s, 2H), 1.54 (s, 6H), 1.35 (s, 18H), 1.21-1.20 (m, 12H).

^{13}C NMR (100 MHz, Chloroform-*d*): δ 205.00, 205.00, 171.06, 171.06, 169.73, 169.73, 168.19, 168.19, 167.00, 167.00, 155.20, 155.20, 139.50, 139.50, 137.31, 137.31, 135.05, 135.05, 133.60, 133.60, 130.17, 130.17, 130.17, 130.17, 129.30, 129.30, 128.94, 128.94, 128.94, 128.94, 128.66, 128.66, 128.66, 128.66, 128.27, 128.27, 126.43, 126.43, 126.43, 126.43, 84.17, 84.17, 82.52, 82.52, 81.55, 81.55, 80.68, 80.68, 80.46, 80.46, 78.83, 78.83, 76.43, 76.43, 74.78, 74.78, 74.68, 74.68, 72.12, 72.12, 57.14, 57.14, 57.07, 57.07, 56.80, 56.80, 56.41, 47.35, 47.35, 43.33, 43.33, 34.99, 34.99, 34.08, 34.08, 32.01, 32.01, 30.76, 30.76, 30.76, 30.76, 28.17, 28.17, 28.17, 28.17, 28.17, 28.17, 26.68, 26.68, 24.81, 24.81, 22.81, 22.81, 21.03, 21.03, 14.48, 14.48, 10.37, 10.37.

Synthesis of dimer PPa conjugate

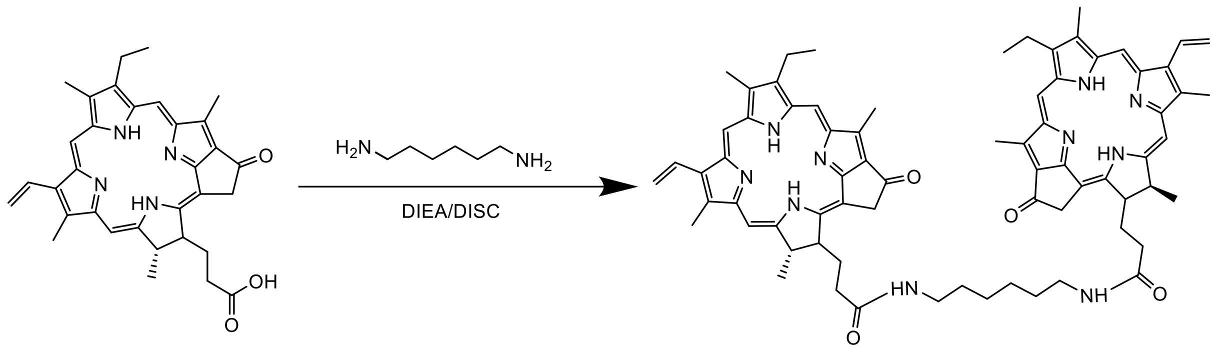

2

1,6-Diaminohexane (7.9 mg, 0.068 mmol, 1.0 eqv.), *N, N*-diisopropylethylamine (DIEA) (19.34 mg, 0.15 mmol, 2.2 eqv.), and DISC (18.9 mg, 0.15 mmol, 2.2 eqv.) were added to a solution of pyropheophorbide-a (PPa, 80 mg, 0.15 mmol, 2.2 eqv.) in 2 mL of dry DCM. The reaction mixture was stirred at 45ºC for 4 h, followed by cooling to room temperature and removal of the solvent. The residue was further dissolved in DCM and was sequentially washed with 5% aqueous citric acid, saturated aqueous $NaHCO_3$ and brine. The organic layer was dried over Na_2SO_4, filtered, and then evaporated under vacuum. Finally, the crude residue was purified by column chromatography (DCM: methanol = 20:1) on silica gel to obtain the dimer conjugate diPPa (73.8mg, 94.5%).

^{1}H NMR (400 MHz, Chloroform-*d*) δ 9.47 (s, 2H), 9.37 (s, 2H), 8.54 (s, 2H), 8.03-7.96 (dd, 2H, *J*1 = 17.8, *J*2=11.5), 6.64 (s, 2H), 6.32-6.14 (m, 4H), 5.30-5.05 (m, 4H), 4.52-4.45 (m, 2H), 4.36-4.32 (m, 2H), 4.13-4.07 (m, 2H), 3.70-3.63 (m, 10H), 3.62-3.54 (m, 2H), 3.40 (s, 6H), 3.22 (s, 6H), 2.77-2.61 (m, 2H), 2.59-2.51 (m, 2H), 2.45-2.36 (m, 2H), 2.27-2.15 (m, 2H), 1.79-1.81 (d, 6H, *J* = 7.3), 1.70-1.67 (t, 6H, *J* = 7.6), 1.15-1.12 (m, 12H).

^{13}C NMR (100 MHz, Chloroform-*d*) δ 196.28, 196.28, 171.52, 171.52, 160.34, 160.34, 155.04, 155.04, 153.75, 153.75, 150.61, 150.61, 148.86, 148.86, 144.82, 144.82, 141.45, 141.45, 137.68, 137.68, 136.05, 136.05, 135.91, 135.91, 135.68, 135.68, 131.47, 131.47, 130.31, 130.31, 129.11, 129.11, 128.09, 128.09, 122.45, 122.45, 106.01, 106.01, 103.85, 103.85, 97.01, 97.01, 92.93, 92.93, 51.61, 51.61, 49.94, 49.94, 48.07, 47.49, 42.64, 42.64, 23.18, 23.18, 22.05, 22.05, 21.93, 21.93, 20.81, 20.81, 20.70, 20.70, 19.33,19.33, 17.42, 17.42, 12.11,12.11, 11.93, 11.93, 11.12, 11.12.

Molecular dynamics simulations

The structures of diCTX and diPPa were optimized using Gaussian16 under B3LYP/6-31G(d) level. Partial atomic charges were computed based on the QM ESP results employing the RESP charge methodology. To initialize the simulations, a mixture of 15 diCTX molecules and 30 diPPa molecules was randomly packed into a cubic box with a length of 120 Å, with the remaining space filled by 20,000 water molecules, using PACKMOL2.

All molecular dynamics (MD) simulations were conducted using Amber22. The Particle Mesh Ewald method with a cutoff of 10 Å was applied to account for long-range electrostatic interactions. Before the heating and equilibrium stages, the system underwent relaxation via a 10,000-step conjugate gradient method and a 10,000-step steepest descent method. During the production run, an integration time step of 2 fs was employed to integrate the equations of motion, and the simulation temperature was set at 300 K. In the production phase, the density was adjusted to 1 dm/L, and a 500-ns simulation under the NVT ensemble was executed.

Evaluation of noncovalent interactions in nanoparticle assembly

The possible noncovalent interactions in forming cabazitaxel and pyropheophorbide a (PPa)-formulated nanoassemblies (CPNA) were investigated using solutions containing Triton X-100, urea, or sodium chloride. Briefly, a solution of mixed dimeric prodrugs in dimethyl sulfoxide (DMSO) was ultrasonically introduced into deionized (DI) water with varying concentrations of Triton X-100, urea, and sodium chloride (0, 1, 5, 10, 25, and 50 mM). The formation of nanoassemblies was monitored by assessing changes in hydrodynamic diameters (D_H) and polydispersity index (PDI). The solutions were stored at 25°C, and particle size distribution was determined through dynamic light scattering (DLS) measurements.

DLS analysis and morphology study

D_H, PDI, and zeta potentials of different nanoassemblies were determined using DLS measurement with a Malvern Nano-ZS90 instrument (Malvern Instruments, Malvern, UK) at 25°C.

The morphologies of the bare and PEGylated nanoassemblies were examined via transmission electron microscopy (TEM). Accordingly, nanoassembly solutions at a concentration equivalent to 0.1 mg/mL cabazitaxel were deposited onto 300-mesh carbon-coated copper grids for 2 min. Subsequently, any residual liquid on the surface of the copper grid was removed using filter paper, and the samples were stained using a 2 wt% aqueous uranyl acetate solution at 25°C for 1 min. After air-drying, morphological characterization was conducted using TEM (Tecnai G2 Spirit, Thermo FEI) at an acceleration voltage of 120 kV.

Optimization of CPNA constructed from diCTX and diPPa conjugates

To determine the optimal dose ratios of diCTX and diPPa, two parameters, particle size and combination index (CI), were included. The D_H, distribution, and zeta potentials of CPNA were evaluated at different molar ratios of diCTX to diPPa, ranging from 10/1 to 1/5 using a Malvern Nano-ZS90 instrument at 25°C. Pure prodrug nanoassemblies were included as a control.

To assess the effects of different proportions on cell viability, CI values were determined in B16F10 cells. Cells (1.5×10^3 cells/well) were seeded in 96-well plates and cultured for 24 h. Subsequently, the culture medium was replaced with fresh medium containing varying concentrations of nanotherapeutics and incubated for 12 h. Subsequently, the drug-containing medium in each well was replaced with a fresh culture medium, and the cells were subjected to a 660 nm laser for 5 min (300 mW/cm^2). After another 60 h of incubation, cell viability was assessed using a cell counting kit-8 (CCK-8) assay and quantified with a microplate reader (Multiskan FC, Thermo Scientific) at 450 nm. Furthermore, CI values were calculated using CompuSyn software, following the Chou-Talalay method, which provides a quantitative definition for different effects: additive (CI = 1), synergistic (CI < 1), and antagonistic (CI > 1). A lower CI typically indicates enhanced synergy in the drug combination.

In vitro stability evaluation

The stability of bare nanoassemblies and CPNA (0.1 mg/mL cabazitaxel-equivalent concentration) in DI water or DI water containing 10% (v/v) fetal bovine serum (FBS) at 37°C was evaluated by monitoring the variation of D_H and PDI. DLS analysis was employed to measure the particle sizes and PDIs of CPNA at predetermined time points during a 7-day incubation.

The diCTX nanoassemblies, diPPa nanoassemblies and CPNA (0.1 mg/mL cabazitaxel-equivalent concentration and 0.13 mg/ml PPa-equivalent concentration) were incubated in DI water at 37°C. The properties of the nanoparticle solutions were monitored until visible precipitates formed in one of the samples.

Measurement of critical micelle concentration

To determine the critical micelle concentration (CMC) of CPNA, a series of nanoassemblies derived from diCTX and diPPa prodrugs at concentrations ranging from 6.1 × 10^{-6} to 0.025 mg/mL (cabazitaxel equivalence) were prepared. The scattering intensity of the samples was measured using a Malvern Nano-ZS90 instrument (Malvern, UK) at 25°C. The intensity data were processed and plotted as a function of the logarithm of the micelle concentration. The CMC value was determined as the point where the two tangents on the curves intersected.

ROS generation potential in aqueous solutions

The ROS generation potentials of free PPa, diPPa nanoassemblies, and CPNA were assessed using UV-vis spectrometry with the ROS capture agent indocyanine green (ICG). Aqueous solutions of different formulations, each with an equivalent PPa concentration of 16 μg/mL, were mixed with ICG (16 μg/mL) and exposed to 660-nm laser irradiation (300 mW/cm^2) for varying time intervals. An ICG solution without PPa, subjected to the same irradiation, was used as a control group. The rate of ROS generation was determined by measuring the decrease in ICG absorption at 779 nm using a UV-vis spectrometer (Shimadzu, UV-2700) at 25°C.

In vitro intracellular uptake by macrophages

Raw264.7 mouse macrophage cells were seeded at a density of 2 × 10^5cells/well in 12-well plates and incubated overnight for adhesion DiI labeled nanoassemblies PEGylated with varying weight percentage (wt%) of LA_2-PEG_{2K} (i.e., 0%, 5%, 10%, 20%, 50%) were added to each well at a concentration of 100 nmol/L DiI and incubated for 1 h, 3 h, and 6 h at 37°C. Control cells received no nanoparticle treatment. After incubation, the culture media containing the nanoparticles were removed, and the cells were washed with cold PBS. Subsequently, the cells were harvested, resuspended in PBS, and cellular uptake of the nanoparticles was quantified using flow cytometry.

Calcein-acetoxymethyl ester/propidium iodide staining following treatment with various hybrid nanoformulations

B16F10 cells and A375 cells were seeded into flat-bottom 12-well plates with 6-8 × 10^4 cells/well and incubated overnight at 37°C to allow for adherence. Cells were then treated with a combination of nanoparticles (diCTX nanoassemblies + diPPa nanoassemblies), bare coassembled nanoparticles, or CPNA, all at a 25 nM cabazitaxel-equivalent concentration. After 12 h of incubation, the drug-containing medium was replaced with fresh culture medium, and the cells were exposed to a 660-nm laser for 5 min (300 mW/cm^2). Following another 2-h incubation, the medium was replaced with calcein-AM and PI staining solutions, and the cells were incubated in the dark for 30 min. Finally, the stained cells were imaged under fluorescence microscopy (Olympus, IX71).

Flow cytometry analysis for cell apoptosis induced by various hybrid nanoformulations

2 × 10^5 B16F10 cells/well and 2.5 × 10^5 A375 cells/well were seeded in flat-bottom 6-well plates and cultured for adherence overnight. Subsequently, cells were exposed to different hybird nanoformulations at a 75 nM cabazitaxel-equivalent concentration. After 12 hours of treatment, the medium was refreshed, and the cells were irradiated with a 660-nm laser (300 mW/cm^2) for 5 min. following an additional 60-h incubation, the cells were collected and costained with an Annexin V-FITC and PI detection kit as per the manufacturer's protocol. Finally, early and late apoptotic cells were quantified using flow cytometry within 1 h.

In vivo evaluation of anti-PEG antibody expression

The level of anti-PEG antibody induced by PEGlayted nanoparticles was evaluated in healthy C57BL/6 mice aged 4-5 weeks. Mice were randomly divided into three groups (n = 4 per group) and received intravenous injections of either PEG_{2K}-PCL_{2K} micelles or LA_2-PEG_{2K} micelles (PEG equivalent dose of 5 µmol/kg) on days 0 and 3. Blood samples (100-150 µL) were collected in EP tubes on days 0, 4, 7, and 14. After allowing the blood to clot for 2 hours, the samples were centrifuged at 3000 × g for 20 min to obtain serum. The levels of anti-PEG IgG and IgM were measured using ELISA. PEG_{2K}-PCL_{2K} and LA_2-PEG_{2K} were dissolve in anhydrous ethanol at a concentration of 0.2 µmol/mL, and 50 µL of solutions were added per well in a 96-well microplate and left to dry overnight at room temperature. The next day,100 µL of blocking solution (PBS with 1% bovine serum albumin) was added to each well, followed by a 1-h incubation. The wells were then washed three times with washing buffer (PBS containing 0.1% bovine serum albumin). Diluted serum samples (1:100, v/v) were added to the microplate at 100 µL per well, with wells lacking serum samples serving as blank controls. Following 2 h of incubation, the microplate was washed three times. Subsequently, HRP-conjugated Affinipure Goat Anti-Mouse IgM or HRP-conjugated Affinipure Goat Anti-Mouse IgG (Proteintech Group, Inc.) were added and incubated for 1 h at room temperature. After washing thrice, TMB substrate solution (Beyotime Biotechnology, China) were added and incubated in the dark for 20 minutes. Finally, TMB stop solution was added, and absorbance was measured at 450 nm using a microplate reader.

Evaluation of in vivo CPNA biodistribution in human melanoma tumors in mice

BALB/c nude mice were subcutaneously inoculated with 2 × 10^6 A375 cells per mouse in the right flank. Once tumors reached approximately 500 mm^3 in the volume, mice were randomly divided into two groups (n = 5 per group). To trace the *in vivo* biodistribution of CPNA, the NIR fluorescent dye DiR was co-assembled into the nanoassemblies to form DiR labeled CPNA (1mg/kg DiR, 6.0 mg/kg cabazitaxel, and 7.7 mg/kg PPa). The CPNA-treated group received a single tail vein injection of DiR labeled CPNA, while the control group received free DiR (1 mg/kg, dissolved in polysorbate 80/ethanol, 1:1, v/v). After 24 h, the mice were euthanized and major tissues (heart,

liver, spleen, lung, kidney) and tumors were collected for *ex vivo* NIR fluorescence imaging using a multimodal animal live imaging system (AniView 100, BLT, China). Tumor samples were then processed into frozen sections using a histological frozen slicer (HM550 vpd, Thermo Fisher Scientific, USA). The slices were fixed with methanol and stained with 4,6-diamidino-2-phenylindole (DAPI) for 10-15 minutes at room temperature. Finally, the sealed sections were imaged under a confocal laser scanning microscope (CLSM, FV3000, Olympus, Japan).

Efficacy testing against a preclinical human melanoma mouse model

A xenograft model was established using BALB/c nude mice injected subcutaneously with 2 × 10^6 human melanoma A375 cells in the right flank. After tumor establishment (approximately 10 days post-injection) with tumors reaching a volume of ~100 mm^3, the mice were randomly divided into seven groups (n = 7 per group). The groups received different treatments via intravenous administration every 3 days for a total of 3 doses, including saline, diCTX nanoassemblies (6.0 mg/kg cabazitaxel, without NIR laser), diPPa nanoassemblies (7.7 mg/kg PPa) with and without NIR laser), low dose CPNA (6.0 mg/kg cabazitaxel and 7.7 mg/kg PPa) with and without NIR laser, and high dose CPNA (18.0 mg/kg cabazitaxel and 23.1 mg/kg PPa) with NIR laser. At 6 h post-injection, the tumor tissues in the laser irradiation group received a localized irradiation with a 660 nm laser at an intensity of 600 mW/cm^2 for a duration of 10 min. Tumor volume and body weight were monitored, and photographs of the tumors were taken on days 0, 7, and 14 post-administrations. The tumor volume was calculated with the formula: $V = (L \times W^2) / 2$ (L: length, W: width). The mice were euthanized once the tumor volume exceeded 2000 mm^3. Additionally, tumor tissues from each group were excised on day 9 post-treatment, fixed with 4% formaldehyde, and subjected to histopathological analysis using H&E staining, TUNEL staining, and Ki67 immunohistochemistry. The tissue slices were visualized using a fluorescence microscope (Olympus, IX71).

Mice bearing A375 tumors (~200 mm^3) were randomly divided into three groups (n = 8 per group). The groups received intravenous tail vein injections every 3 days for a total of 3 doses: saline, diCTX nanoassemblies followed by diPPa nanoassemblies with NIR laser, and CPNA with NIR laser, at dose of 12.0 mg/kg cabazitaxel and 15.4 mg/kg PPa. 6 h post- administration, the tumor tissues were irradiated with a 660 nm laser at an intensity of 600 mW/cm^2 for a duration of 10 min. Tumor volume and body weight were monitored ever two days.

Evaluation of systemic toxicity and safety profiles

The systemic toxicity of CPNA and a free drug combination was evaluated in healthy ICR mice aged 4-5 weeks. Mice were randomly divided into three groups (n = 5 per group) and received three intravenous doses every 3 days of either CPNA nanoassemblies (27.0 mg/kg cabazitaxel and 34.8 mg/kg PPa), the free drug combination (9.0 mg/kg cabazitaxel and 11.6 mg/kg PPa), or saline as a control. Mouse body weight changes were monitored for 20 days post-injection. Blood samples

(100-150 μL) were collected in EDTA anticoagulant tubes on days 0, 3, 5, and 7 to analyze hematological parameters, including neutrophils (NEs), white blood cells (WBCs), lymphocytes (LYs) and red blood cells (RBCs) counts, to assess potential hematological side effects such as leukopenia and neutropenia.

Blood biochemistry analyses were conducted to assess C-reactive protein (CRP) levels and markers of hepatic and renal function in the serum. Blood samples were collected on day 8 post-administration and centrifuged at 3000 × g for 20 min to obtain serum. CRP levels were measured using the Mouse C-Reactive Protein (CRP) ELISA Kit (Servicebio®). Hepatorenal parameters in the serum, including hepatic function markers (alanine aminotransferase, ALT; and aspartate aminotransferase, AST) and renal function markers (blood urea nitrogen, BUN; creatinine, CR; and uric acid, UA) were analyzed after the treatment.

Finally, the immunotoxicity of CPNA and the free drug combination was assessed in healthy ICR mice using the same administration regimen. After three times of drug administration, the major organs of experimental mice (heart, liver, spleen, lung, and kidney) of the experimental mice were excised and weighed. The tissues were then homogenized in RIPA lysis buffer at 4°C using a high-speed tissue homogenizer (Servicebio, KZ-II). The tissue homogenate was diluted by PBS for testing IL-6, IL-12, and TNF-α levels using ELISA kit.

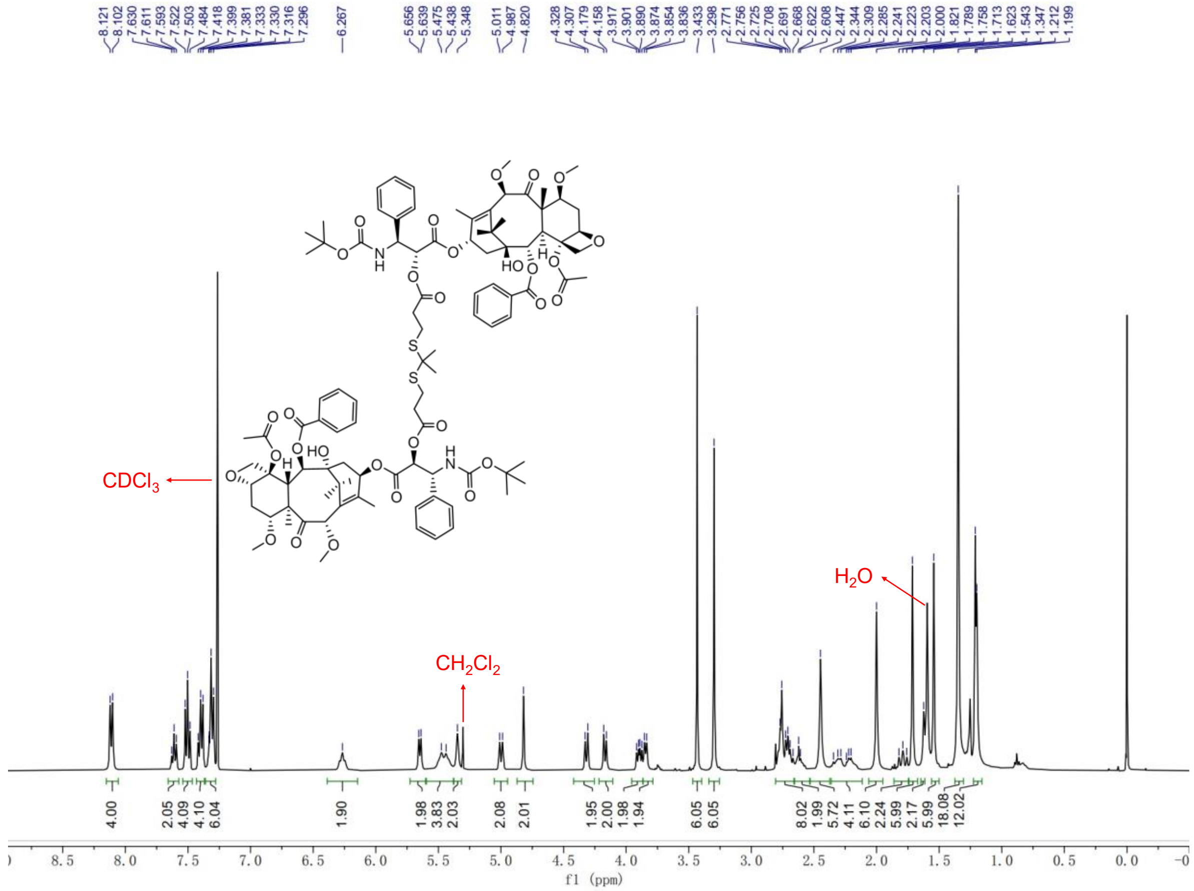

Figure S1. ^{1}H NMR spectrum of diCTX in $CDCl_3$.

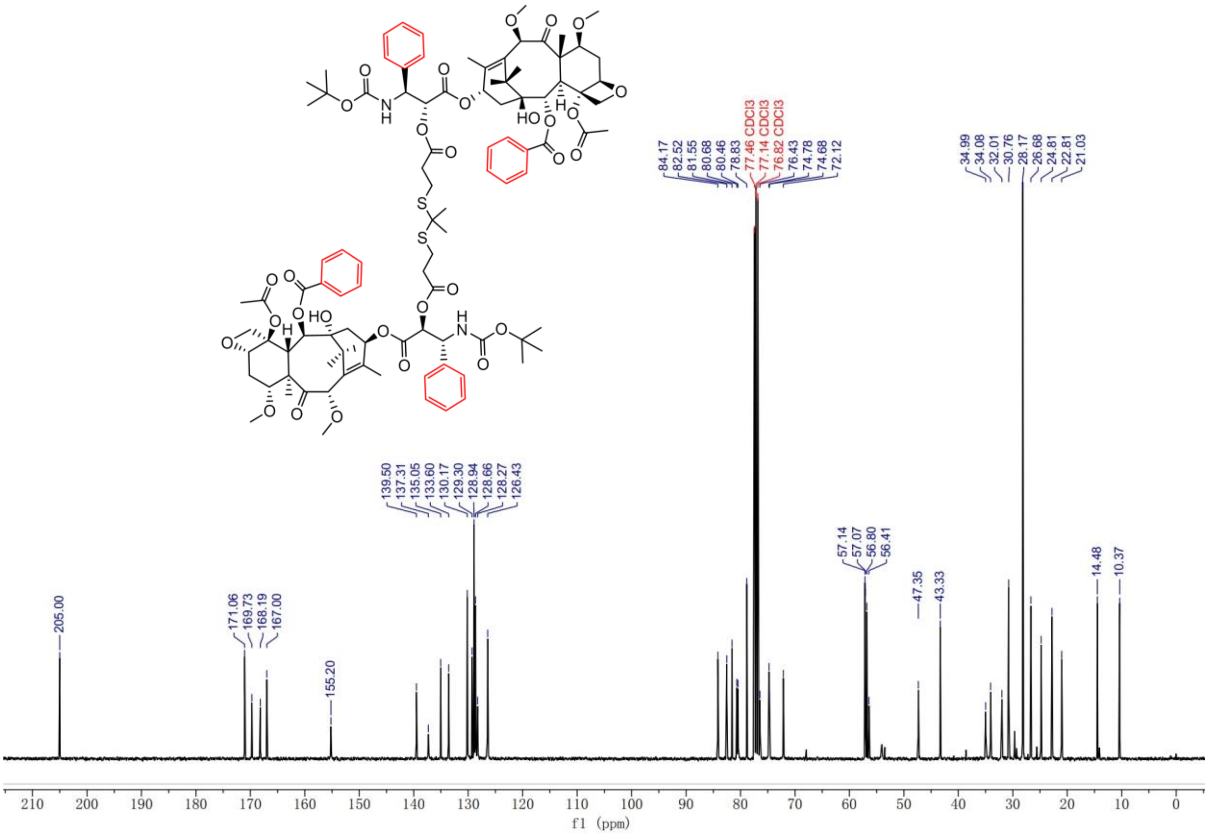

Figure S2. ^{13}C NMR spectrum of diCTX in $CDCl_3$.

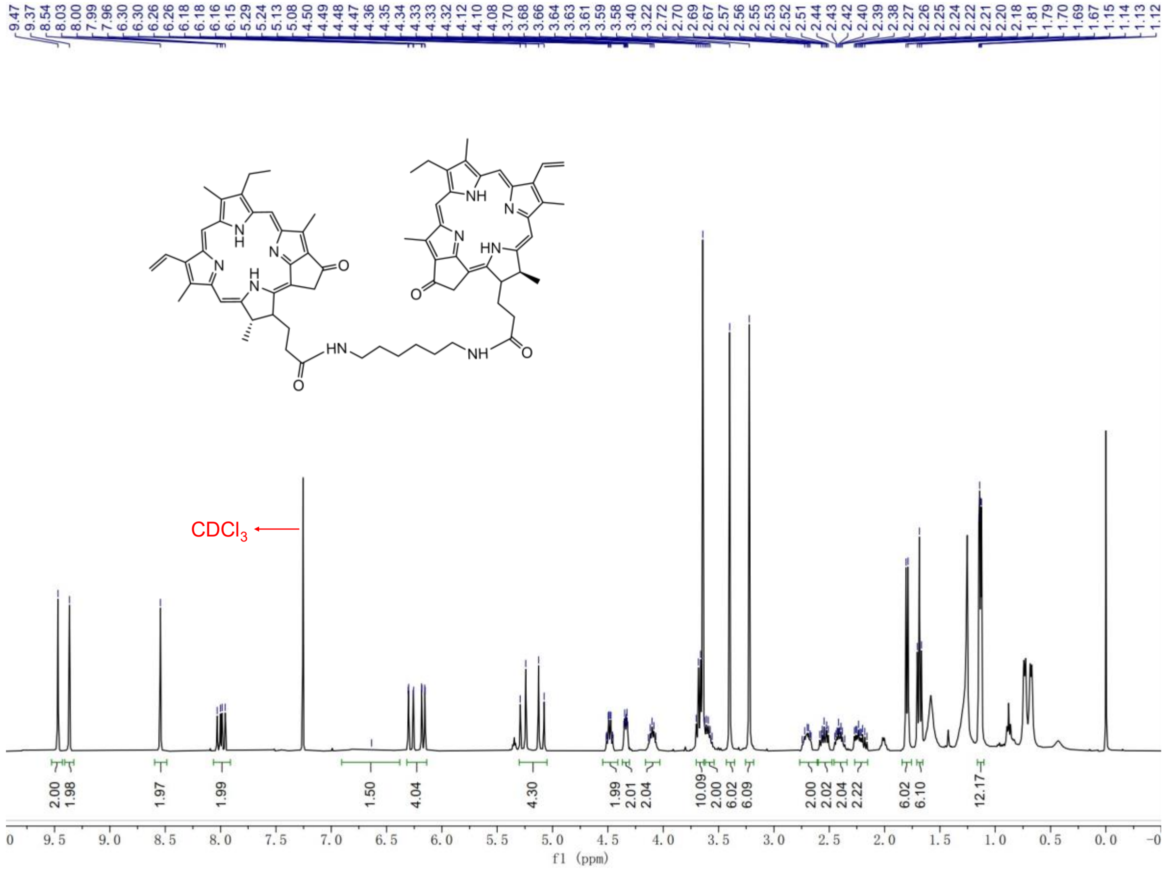

Figure S3. ^{1}H NMR spectrum of diPPa in $CDCl_3$

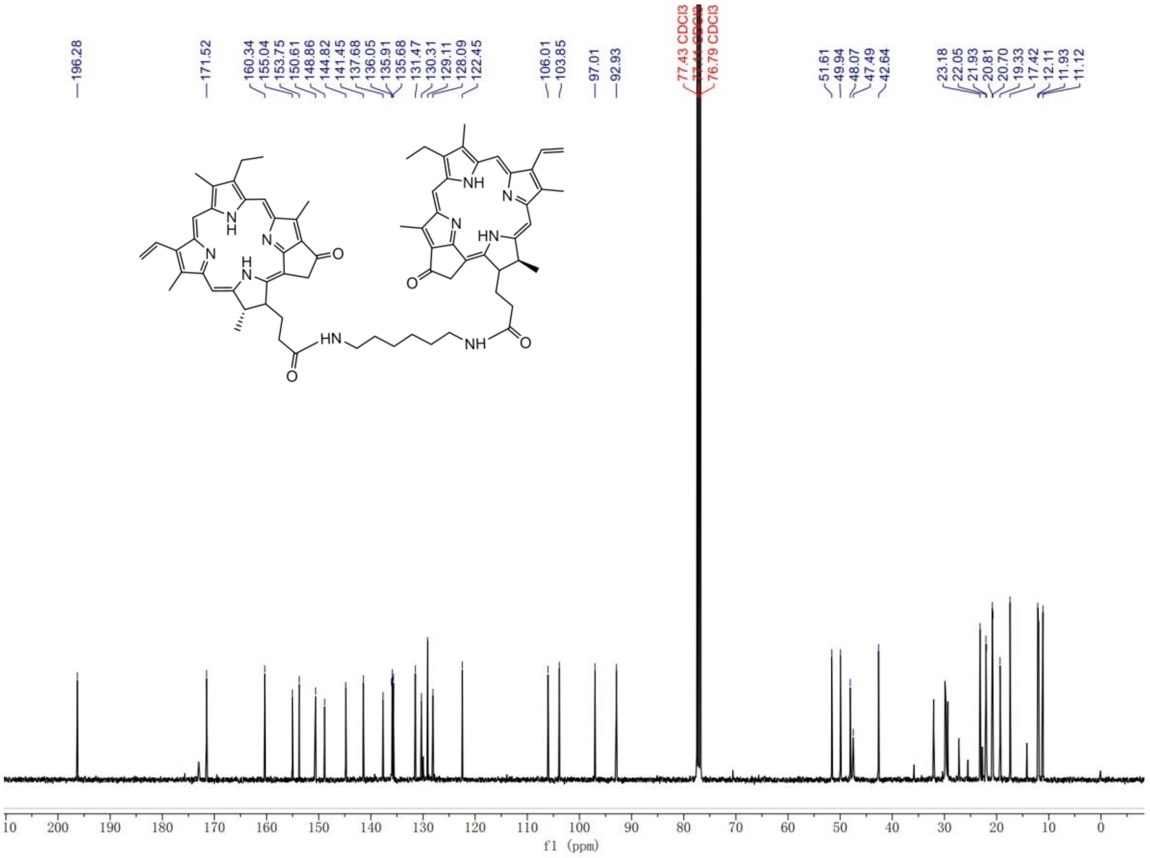

Figure S4. ^{13}C NMR spectrum of diPPa in $CDCl_3$.

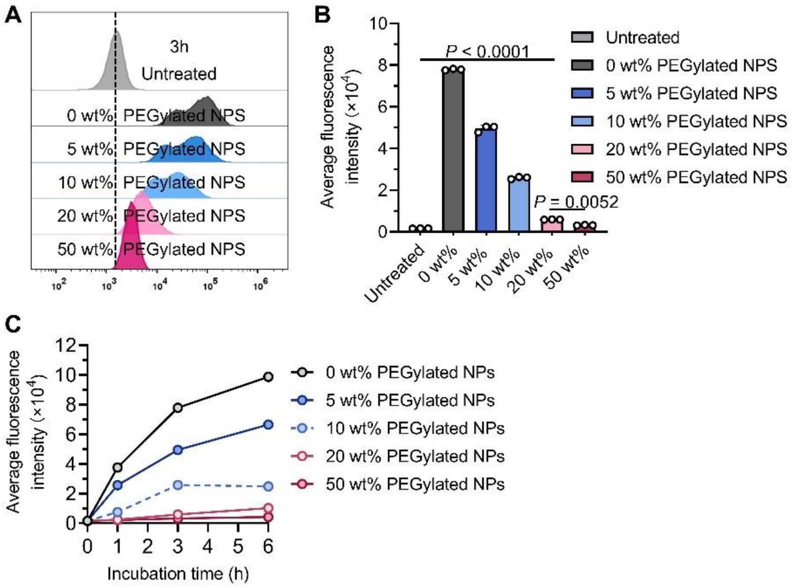

Figure S5. Intracellular uptake of DiI labeled nanoassemblies by RAW264.7 Cells. (A and B) Flow cytometry analysis of the uptake of DiI-labeled nanoassemblies by mouse macrophage RAW264.7 cells after 3 h of incubation at 37°C (n = 3). Untreated cells were used as the negative control. (C) Quantification of the intracellular uptake of various DiI-labeled nanoassemblies after 1 h, 3 h, and 6 h of incubation at 37°C, as measured by flow cytometry (n = 3). Data are presented as the mean ± standard deviation. Statistical significance was evaluated using one-way ANOVA (B) followed by Tukey's multiple comparisons test.

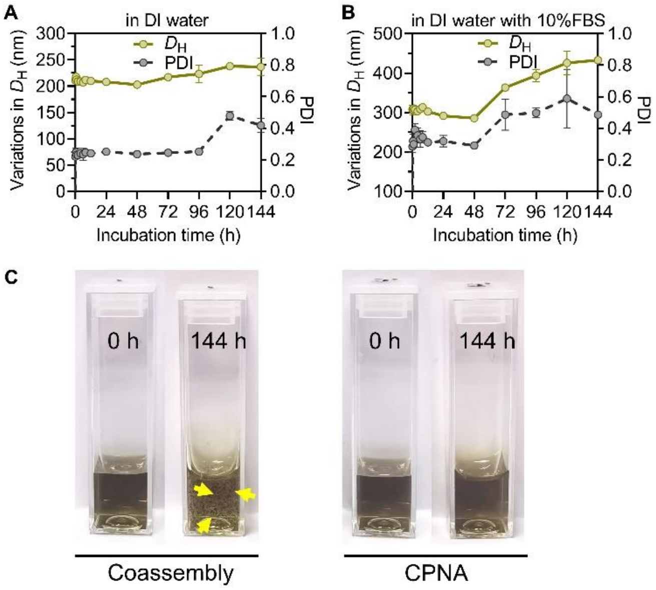

Figure S6. Stability evaluation of non-PEGylated bare coassembled nanoassemblies. (A and B) Analysis of the s tability of non-PEGylated bare coassembled nanoassemblies through changes in hydrodynamic diameters (D_H) and polydispersity index (PDI) in deionized (DI) water

and DI water containing 10% (v/v) fetal bovine serum (FBS). (C) Photographs of t the solution of bare nanoparticles (left) and CPNA (right) in DI water after 144 h of storage.

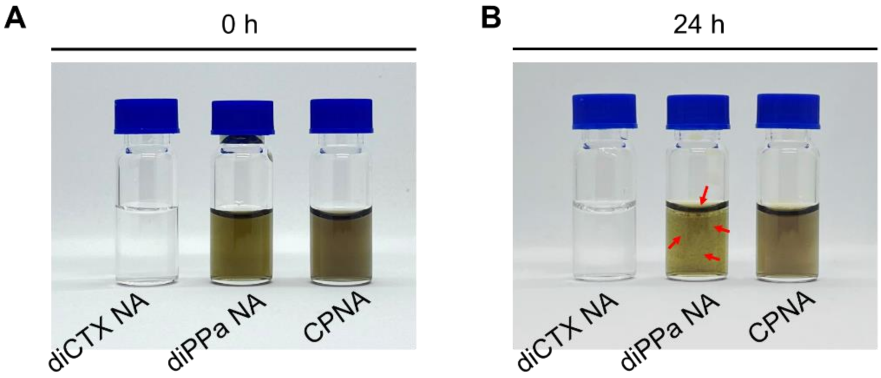

Figure S7. Stability evaluation of nanoassemblies constructed from individual prodrugs. Photographs of the solutions of diCTX nanoassemblies (diCTX NA, left), diPPa nanoassemblies (diPPa NA, middle), and CPNA (right) in DI water were taken initially (A) and after 24 h of storage (B).

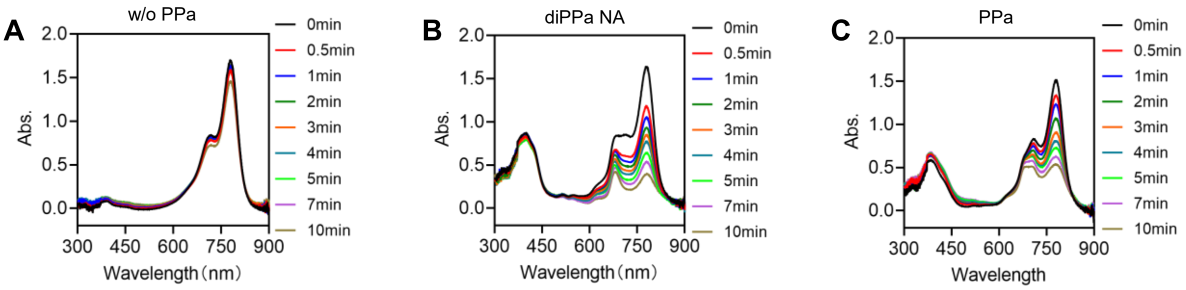

Figure S8. Time-dependent absorption spectra of ICG in the absence (A) or presence of diPPa nanoassemblies (B) or free PPa (C) upon 660 nm laser irradiation (300 mW/cm^2).

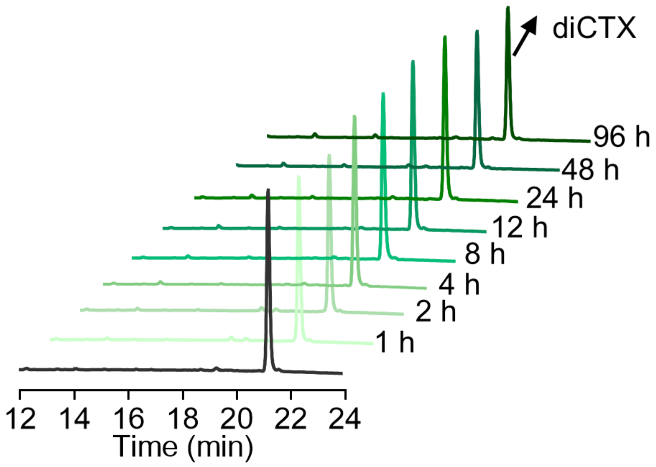

Figure S9. Representative HPLC chromatograms of the diCTX prodrug in the absence of H_2O_2.

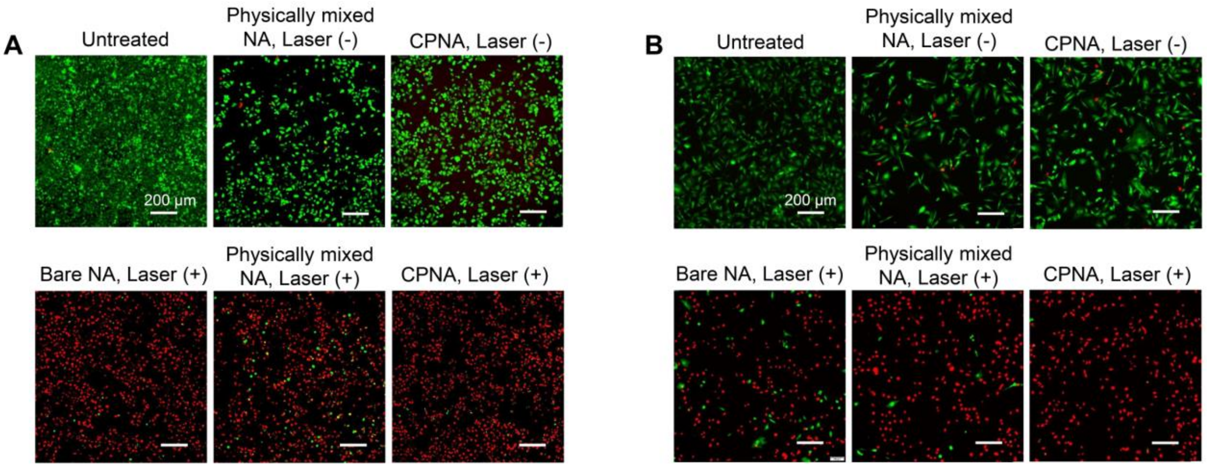

Figure S10. Fluorescence microscopy images revealing dead/live cell staining of A375 cells (A) and B16F10 cells (B). Cells were treated with drugs (physically mixed diCTX and diPPa nanoassemblies (NA), bare coassembled nanoassemblies (NA), and CPNA) at a cabazitaxel-equivalent concentration of 25 nM. Following laser irradiation (660 nm, 300 mW/cm^2, 5 min), the cells were co-stained with calcein-AM (green, live cells) and PI (red, dead cells). Scale bars, 200 µm.

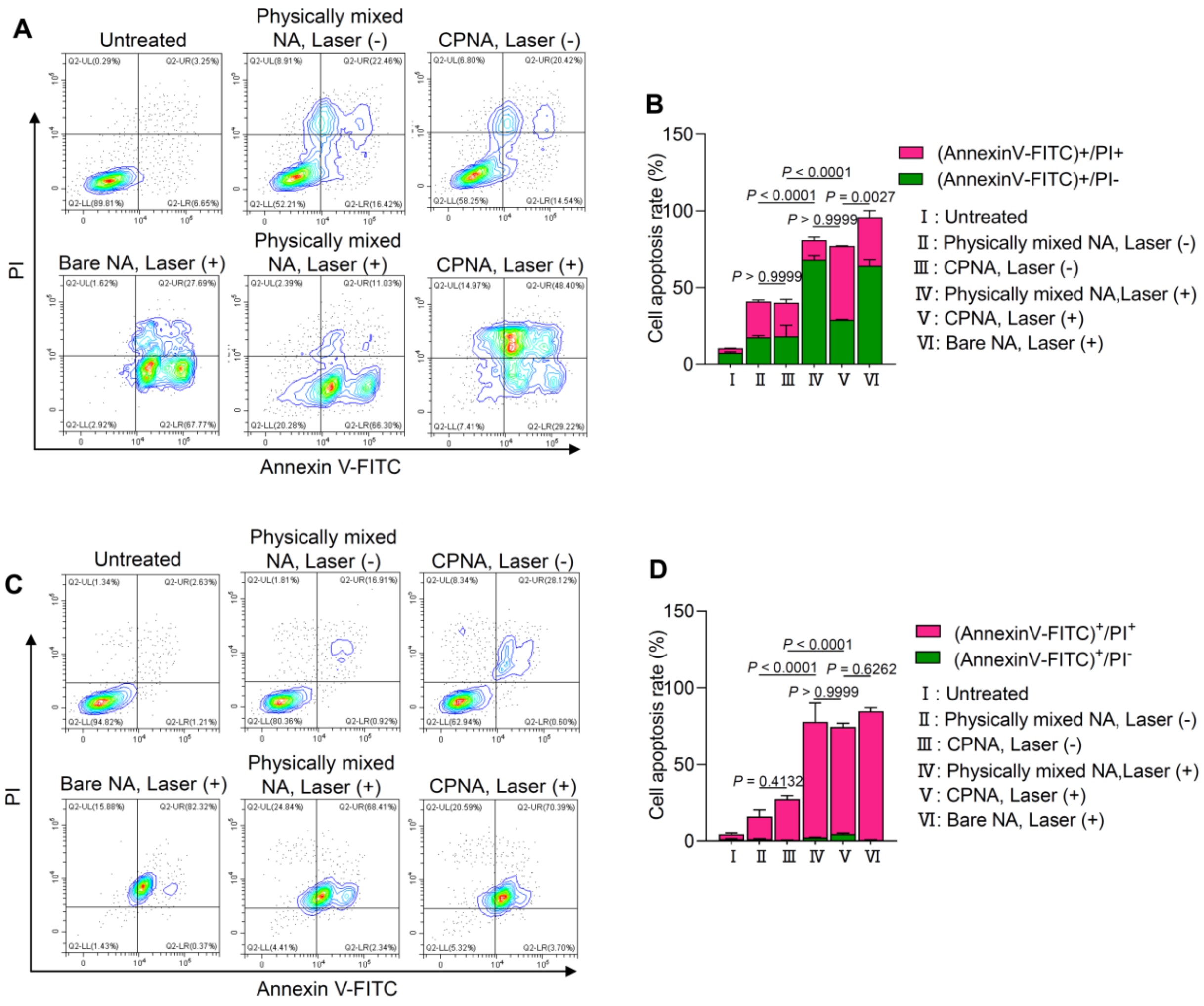

Figure S11. Flow cytometry analysis of A375 (A-B) and B16F10 (C-D) apoptosis following different treatments (physically mixed diCTX and diPPa nanoassemblies (NA), bare coassembled nanoassemblies (NA), and CPNA) at a cabazitaxel-equivalent concentration of 75 nM and PPa-equivalent concentration of 150 nM (n = 3). Cells were treated with hybrid nanoformulations, irradiated with a laser (660 nm, 300 mW/cm^2, 5 min), and costained using an Annexin V-FITC and PI detection kit. Data are presented as mean ± standard deviation. Statistical significance was evaluated using one-way ANOVA (B and D) followed by Bonferroni's multiple comparisons test.

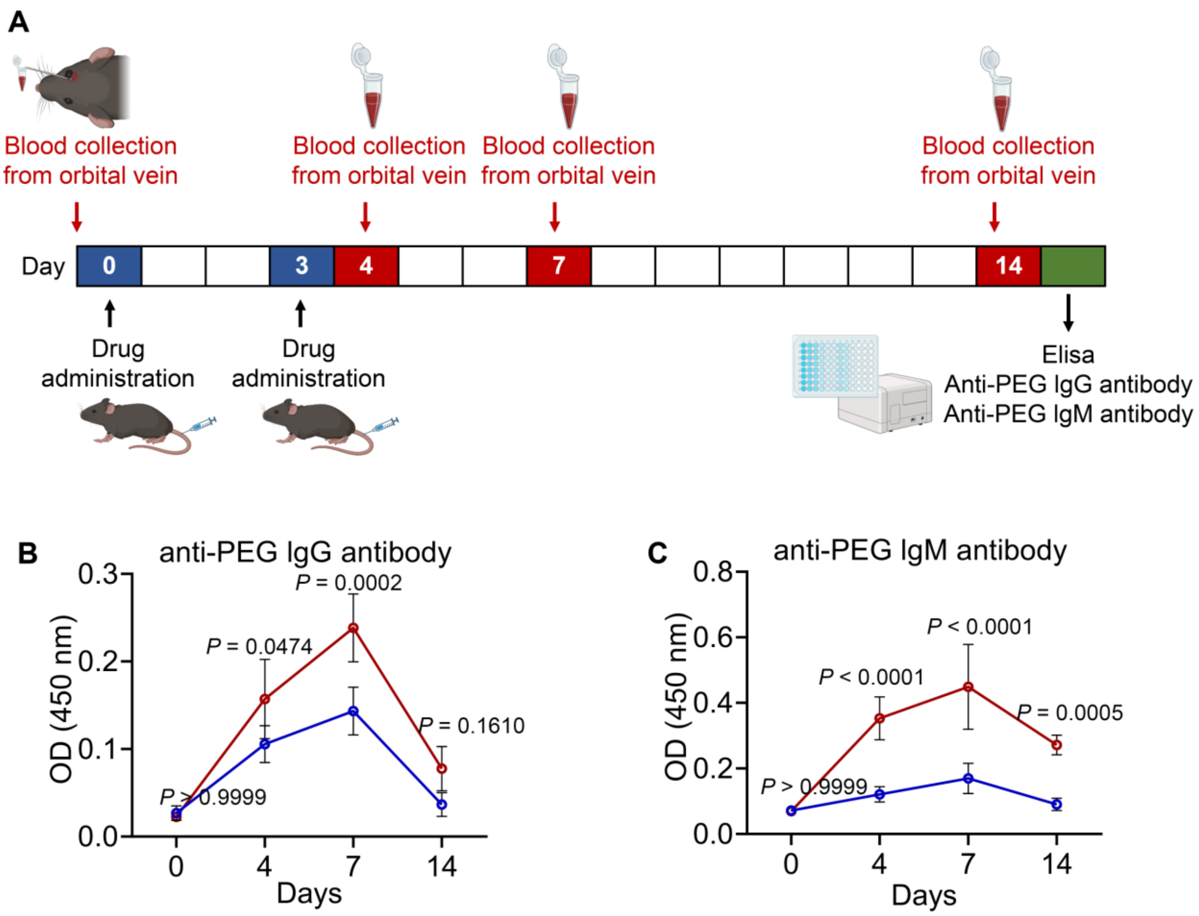

Figure S12. ELISA analysis of anti-PEG antibody induced by PEGylated nanoparticles. (A) Schematic illustration of the experimental timeline for assessing anti-PEG antibody expression. Illustration created with BioRender.com. (B and C) Change curves of anti-PEG IgG and IgM antibody levels on days 0, 4, 7, and 14 post-administration (n = 4). Red line: PEG_{2K}-PCL_{2K}; blue line: LA_2-PEG_{2K}; Data are presented as mean ± standard deviation. Statistical significance was evaluated using two-way ANOVA (B and C) followed by Bonferroni's multiple comparisons test.

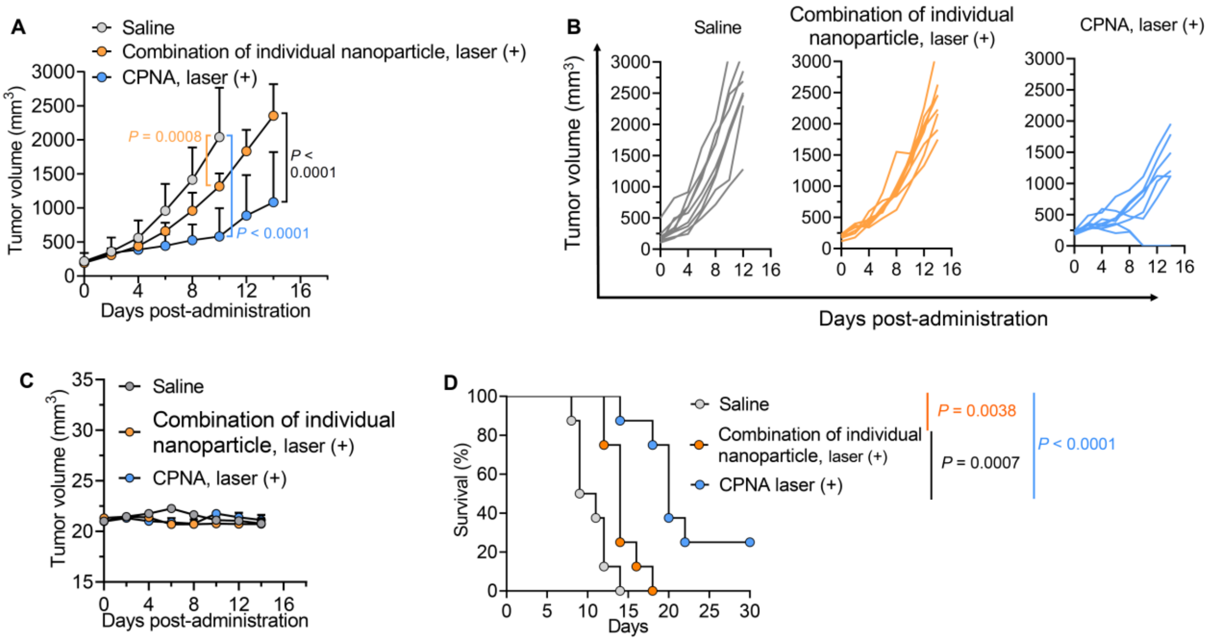

Figure S13. Antitumor efficacy of the combination of individual diCTX and diPPa nanoparticle in an A375 melanoma-bearing mouse model compared to CPNA. (A) Tumor growth curves of mice in each group (n = 8). (B) Individual tumor growth curves for mice indicated in A. (C) Monitoring of body weight changes of mice in each group (n = 8). (D) Mouse survival curves of different treatment groups (n = 8). Data are presented as mean ± standard deviation. Statistical significance was evaluated using two-way ANOVA (A) followed by Tukey's multiple comparisons test or log-rank test (J).

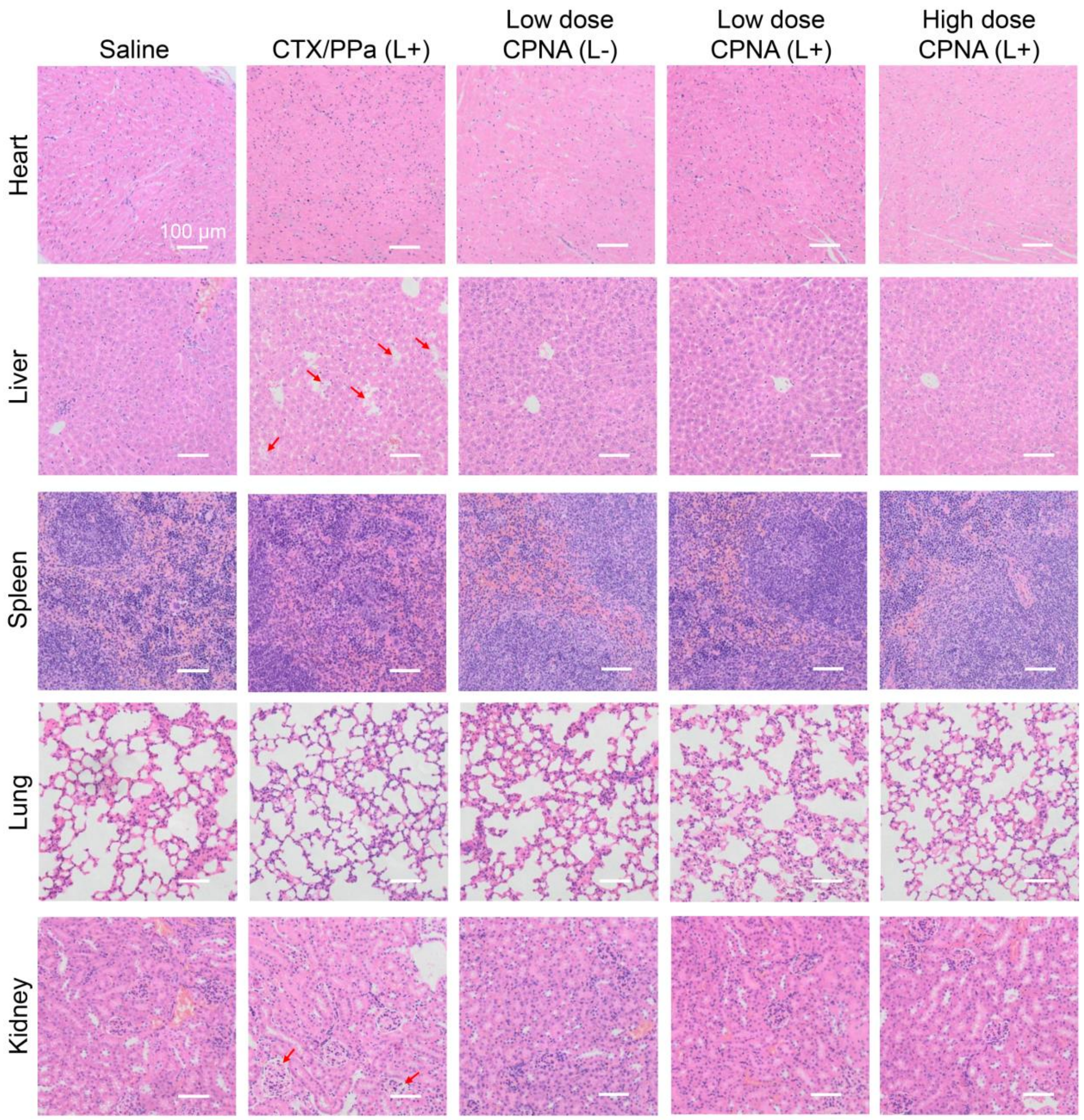

Figure S14. Histological analysis of organ damage in mice receiving various treatments. (A) Representative images illustrating H&E staining of major organs (heart, liver, spleen, lung, and kidney) following treatments, including saline, the combination of free cabazitaxel (3 mg/kg) and PPa (3.9 mg/kg) with laser irradiation, low-dose CPNA (6.0 mg/kg cabazitaxel; 7.7 mg/kg PPa) with or without laser irradiation, and high-dose CPNA (18.0 mg/kg cabazitaxel; 23.1 mg/kg PPa) with laser irradiation.

Table S1. IC_{50} values were extrapolated from dose-response curves (Figure 6A). Cells were treated with CPNA followed by NIR laser irradiation (660 nm, 300 mW/cm^2, 5 min) or without irradiation, and the cell viability was determined by the CCK-8 assay. The data are presented as the means ± SD (n = 3) in nM.

Cells	IC_{50} (nM, PPa)	
	CPNA L (-)	CPNA L (+)
A375	75.1±3.3	55.4±0.8
4T-1	>10000	130.5±3.2
B16F10	334.9±17.8	96.9±2.1
